# A polyphasic study of Hypoxylaceae (Xylariales, Ascomycota) in Iran: new species and records from Hyrcanian forests

**DOI:** 10.3897/mycokeys.131.186541

**Published:** 2026-04-08

**Authors:** Mohammad Javad Pourmoghaddam, Christopher Lambert, Hermann Voglmayr, Seyed Akbar Khodaparast, Marc Stadler

**Affiliations:** 1 Department of Plant Protection, Faculty of Agricultural Sciences, University of Guilan, Rasht, Iran University of Guilan Rasht Iran; 2 Microbial Drugs, Helmholtz-Centre for Infection Research GmbH, Braunschweig, Germany Microbial Drugs, Helmholtz-Centre for Infection Research GmbH Braunschweig Germany; 3 Institute of Microbiology, Technical University Carolo-Wilhelmina Braunschweig, Braunschweig, Germany University of Vienna Wien Austria; 4 Department of Botany and Biodiversity Research, University of Vienna, Wien, Austria Technical University Carolo-Wilhelmina Braunschweig Braunschweig Germany

**Keywords:** Chemotaxonomy, molecular phylogenetics, polyphasic taxonomy, systematics

## Abstract

In a survey of pyrenomycetes in the north of Iran, a total of 20 specimens were collected and characterized via an integrative approach. Sequences derived from the ITS, LSU, *rpb2*, and *tub2* loci were amplified, sequenced, and analyzed in a molecular phylogenetic framework using maximum likelihood and maximum parsimony analyses. Additional morphological and chemotaxonomic evidence analyzed in tandem revealed two new species, which we coined *Hypoxylon hyrcanense***sp. nov**. and *H. pseudoinvestiens***sp. nov**., as well as four new records for Iran, namely *Hypomontagnella submonticulosa*, *Hypoxylon crocopeplum*, *H. larissae*, and *H. ticinense*. We further discuss recent additions to *Hypoxylon* and report the anamorph of *H. larissae* for the first time. All species are illustrated, described, and discussed.

## Introduction

The order Xylariales, first described by Nannfeldt (1932), is one of the largest within the phylum Ascomycota and currently comprises 22 families (Thiyagaraja et al. 2025). It belongs to the subclass Xylariomycetidae within the class Sordariomycetes. Many species in this order are recognized as macromycetes because they often form conspicuous structures, called stromata (Helaly et al. 2018), in which their perithecia, ascogenous structures, are embedded. The stromatic Xylariales can be distinguished by their perithecial stromata, typically dark in color, with thick-walled ascomata containing true paraphyses. They possess periphysate and papillate ostioles, unitunicate asci with eight spores, an inamyloid or amyloid apical apparatus, and pigmented ascospores (Hyde et al. 2020), although morphological characters have repeatedly been shown to deviate from their genetic affinities (Jaklitsch et al. 2016; Cedeño-Sanchez et al. 2023a).

The current study treats the Hypoxylaceae, which was resurrected by Wendt et al. (2018). The current concept of the family integrates multiple characteristics, such as the presence or absence of specific secondary metabolites, anamorph and teleomorph morphology, as well as multi-locus sequencing and phylogenetic inference. Hypoxylaceous species are ubiquitously distributed and are most species-rich in subtropical and tropical regions (Daranagama et al. 2018). Many taxa in the family have been described to occupy multiple niches as saprobes, endophytes, or (rarely) phytopathogens and have even been shown to utilize insects as vectors (Helaly et al. 2018). These fungi are also known as a rich source of bioactive chemical compounds, some of which have shown intriguing potential for application in human and veterinary medicine (Becker and Stadler 2021) (Fig. 19).

Extensive data on the diversity of this family around the globe are still missing, suggesting that there is still much to learn about its distribution patterns and biodiversity. This report is devoted to the characterization and description of new collections showing hypoxylaceous affinities during our collecting programs in the north of Iran. The species were studied using morphological, molecular phylogenetic, and chemotaxonomic descriptors.

## Materials and methods

Fresh material was collected during 2015–2022 from Guilan and Mazandaran provinces (Northern Iran). Parts of corticated branches and trunks bearing Hypoxylaceae stromata were transferred to the laboratory. For details of the specimens used for morphological investigations, see the Taxonomy section below. Dried specimens were deposited in the fungarium of the Department of Plant Protection, Faculty of Agricultural Science, University of Guilan, Guilan, Iran (GUM). Living cultures have been deposited in MUCL (Louvain, Belgium).

Fungal material was studied, isolated, and cultured following the methods described by Pourmoghaddam et al. (2020). Pigment colors were determined as described in the color codes of Rayner (1970). Macrophotographs were obtained with a Keyence VHX-6000 microscope. Light microscopy was performed with a Leica DM1000 light microscope equipped with a Canon digital camera (600D). SEM images of ascospores were recorded using a field-emission scanning electron microscope (FE-SEM Merlin, Zeiss, Germany), in a similar fashion to that reported previously (Kuhnert et al. 2017). Adobe Photoshop CS5 (Adobe Systems, USA) was used for the final editing of acquired images and photographic preparations.

### DNA extraction, PCR, and sequencing

DNA extraction from fresh cultures and amplification of the ITS (nuc rDNA internal transcribed spacer region containing ITS1–5.8S–ITS2), LSU (5’ 1,200 bp of the large subunit nuc 28S rDNA), *rpb2* (partial second largest subunit of the DNA-directed RNA polymerase II), and *tub2* (partial β-tubulin) loci were carried out as described by Wendt et al. (2018).

### Molecular phylogenetic analyses

To reveal the phylogenetic position of the Iranian Hypoxylaceae, the newly generated sequences were compared with published sequences of single accessions for each species retrieved from GenBank. Information on the composition of the sequence matrix, i.e., the used strains, their corresponding sequences, and GenBank accession numbers, can be found in Table 1. This matrix was aligned using the server versions of MAFFT v. 7.490 (www.ebi.ac.uk/Tools/mafft or http://mafft.cbrc.jp/alignment/server/; Katoh et al. 2019) and checked and refined manually using BioEdit v. 7.0.4.1 (Hall 1999). The final phylogenetic analysis comprised a total of 196 accessions of Hypoxylaceae, including eight taxa from Graphostromataceae (*Biscogniauxia nummularia*, *Graphostroma platystomum*) and Xylariaceae (*Kretzschmaria deusta*, *K. hedjaroudei*, *Nemania hyrcana*, *N. serpens*, *Xylaria arbuscula*, *X. hypoxylon*) that served as the outgroup. The individually aligned loci of ITS, LSU, *rpb2*, and *tub2* were combined into a supermatrix after deletion of ambiguously aligned and gap-rich regions. The final combined data matrix contained a total of 4,247 alignment positions (535 from ITS, 1,298 from LSU, 1,047 from *rpb2*, and 1,367 from *tub2*). To investigate whether the *tub2* single-locus dataset as a barcode marker in Hypoxylaceae contained similar phylogenetic information, we performed the same analyses on it separately as described above.

**Table 1.** Isolates and accession numbers of sequences used in the phylogenetic analyses. Type specimens are labeled with HT (holotype), IT (isotype), ET (epitype), and PT (paratype). Isolates and sequences in bold were isolated or sequenced in the present study.

Species	Strain number	Origin	Status	GenBank accession number				References
				ITS	LSU	*rpb2*	*tub2*	
*Annulohypoxylon annulatum*	CBS 140775	USA	ET	KY610418	KY610418	KY624263	KX376353	Kuhnert et al. (2017; *tub2*); Wendt et al. (2018: ITS, LSU, *rpb2*)
*Annulohypoxylon annulatum*	DSM 107931	USA		MK287534	MK287546	MK287559	MK287572	Sir et al. (2019)
*Annulohypoxylon annulatum*	MUCL 57733	Iran		PX943591	PX943605	PX984354	PX984366	This study
*Annulohypoxylon chuxiongense*	GMB 0220	China	HT	PQ278776	PQ278822	PQ273652	PQ273687	Li et al. (2025)
*Annulohypoxylon cyclobalanopsidisglaucae*	GMB 0242	China	HT	PQ278769	PQ278813	PQ273643	PQ273678	Li et al. (2025)
*Annulohypoxylon fulvum*	MUCL 54622	French Guiana		KX376337	N/A	N/A	KX376354	Kuhnert et al. (2017)
*Annulohypoxylon fusisporum*	UADY 83	Mexico	HT	OR807998	OR807987	OR825472	OR825468	Reyes et al. (2024)
*Annulohypoxylon limushanense*	GMB 0479	China	HT	PQ278761	PQ278805	PQ273635	PQ273670	Li et al. (2025)
*Annulohypoxylon maolanense*	GMB 0232	China	HT	PQ278764	PQ278808	PQ273638	PQ273673	Li et al. (2025)
*Annulohypoxylon michelianum*	CBS 119993	Spain		KX376320	KY610423	KY624234	KX271239	Kuhnert et al. (2017; ITS, *tub2*); Wendt et al. (2018; LSU, *rpb2*)
*Annulohypoxylon moriforme*	CBS 123579	Martinique		KX376321	KY610425	KY624289	KX271261	Kuhnert et al. (2017; ITS, *tub2*); Wendt et al. (2018: LSU, *rpb2*)
*Annulohypoxylon nitens*	MFLUCC 12-0823	Thailand		KJ934991	KJ934992	KJ934994	KJ934993	Daranagama et al. (2015)
*Annulohypoxylon olivaceogriseum*	GMB 0230	China	HT	PQ278766	PQ278810	PQ273640	PQ273675	Li et al. (2025)
*Annulohypoxylon pseudoalbidiscum*	GMB 0235	China	HT	PQ278788	PQ278834	PQ273664	PQ273699	Li et al. (2025)
*Annulohypoxylon rongjiangense*	GMB 0229	China	HT	PQ278780	PQ278826	PQ273656	PQ273691	Li et al. (2025)
*Annulohypoxylon stygium*	MUCL 54601	French Guiana		KY610409	KY610475	KY624292	KX271263	Wendt et al. (2018)
*Annulohypoxylon substygium*	MUCL 51708	Iran	ET	KC968915	N/A	N/A	KC977285	Kuhnert et al. (2014a)
*Annulohypoxylon substygium*	MUCL 57734	Iran		PV522280	PV522289	PV524395	PV524404	Lambert et al. (2025)
*Annulohypoxylon subyungense*	GMB 0226	China	HT	PQ278787	PQ278833	PQ273663	PQ273698	Li et al. (2025)
*Annulohypoxylon terebratum*	CBS 119137	Thailand	HT	DQ631943	DQ840069	DQ631954	DQ840097	Tang et al. (2009; ITS, *rpb2*); Fournier et al. (2010; LSU, *tub2*)
*Annulohypoxylon truncatum*	CBS 140778	Texas	ET	KY610419	KY610419	KY624277	KX376352	Kuhnert et al. (2017; *tub2*); Wendt et al. (2018; ITS, LSU, *rpb2*)
*Annulohypoxylon viridipigmentum*	GMB 0225	China	HT	PQ278784	PQ278830	PQ273660	PQ273695	Li et al. (2025)
*Annulohypoxylon yunnanense*	GMB 0462	China	HT	PQ278779	PQ278825	PQ273655	PQ273690	Li et al. (2025)
*Biscogniauxia nummularia*	MUCL 51395	France	ET	KY610382	KY610427	KY624236	KX271241	Wendt et al. (2018)
*Daldinia andina*	CBS 114736	Ecuador	HT	AM749918	KY610430	KY624239	KC977259	Bitzer et al. (2008; ITS); Kuhnert et al. (2014a; *tub2*); Wendt et al. (2018; LSU, *rpb2*)
*Daldinia bambusicola*	CBS 122872	Thailand	HT	KY610385	KY610431	KY624241	AY951688	Hsieh et al. (2005; *tub2*); Wendt et al. (2018; ITS, LSU, *rpb2*)
*Daldinia caldariorum*	MUCL 49211	France		AM749934	KY610433	KY624242	KC977282	Bitzer et al. (2008; ITS); Kuhnert et al. (2014a; *tub2*); Wendt et al. (2018; LSU, *rpb2*)
*Daldinia childiae*	CBS 122881	France	HT	KU683757	MH874773	KU684290	KU684129	U’Ren et al. (2016; ITS, *rpb2*, *tub2*); Vu et al. (2019; LSU)
*Daldinia concentrica*	CBS 113277	Germany		AY616683	KY610434	KY624243	KC977274	Triebel et al. (2005; ITS); Kuhnert et al. (2014a; *tub2*); Wendt et al. (2018; LSU, *rpb2*)
*Daldinia dennisii*	CBS 114741	Australia	HT	JX658477	KY610435	KY624244	KC977262	Stadler et al. (2014; ITS); Kuhnert et al. (2014a; *tub2*); Wendt et al. (2018; LSU, *rpb2*)
*Daldinia eschscholtzii*	MUCL 45435	Benin		JX658484	KY610437	KY624246	KC977266	Stadler et al. (2014; ITS); Kuhnert et al. (2014a; *tub2*); Wendt et al. (2018; LSU, *rpb2*)
*Daldinia loculatoides*	CBS 113279	UK	ET	AF176982	KY610438	KY624247	KX271246	Johannesson et al. (2000; ITS); Wendt et al. (2018; LSU, *rpb2*, *tub2*)
*Daldinia macaronesica*	CBS 113040	Spain	PT	KY610398	KY610477	KY624294	KX271266	Wendt et al. (2018)
*Daldinia petriniae*	MUCL 49214	Austria	ET	AM749937	KY610439	KY624248	KC977261	Bitzer et al. (2008; ITS); Kuhnert et al. (2014a; *tub2*); Wendt et al. (2018; LSU, *rpb2*)
*Daldinia placentiformis*	MUCL 47603	Mexico		AM749921	KY610440	KY624249	KC977278	Bitzer et al. (2008; ITS); Kuhnert et al. (2014a; *tub2*); Wendt et al. (2018; LSU, *rpb2*)
*Daldinia pyrenaica*	MUCL 53969	France		KY610413	KY610413	KY624274	KY624312	Wendt et al. (2018)
*Daldinia steglichii*	MUCL 43512	Papua New Guinea	PT	KY610399	KY610479	KY624250	KX271269	Wendt et al. (2018)
*Daldinia theissenii*	CBS 113044	Argentina	PT	KY610388	KY610441	KY624251	KX271247	Wendt et al. (2018)
*Daldinia vernicosa*	CBS 119316	Germany	ET	KY610395	KY610442	KY624252	KC977260	Kuhnert et al. (2014a; *tub2*); Wendt et al. (2018; ITS, LSU, *rpb2*)
*Durotheca comedens*	YMJ 90071615	Taiwan	HT	EF026128	N/A	JX507793	EF025613	Ju et al. (2003)
*Durotheca crateriformis*	GMB C0205	China	HT	MH645426	MH645425	MH645427	MH049441	de Long et al. (2019)
*Durotheca guizhouensis*	GMB C0065	China	HT	MH645423	MH645421	MH645422	MH049439	de Long et al. (2019)
*Durotheca rogersii*	YMJ 92031201	Taiwan	HT	EF026127	N/A	JX507794	EF025612	Ju et al. (2007)
*Entonaema cinnabarinum*	CNF 2/11046	Croatia		OQ863621	OQ863622	OQ877102	OQ877113	Pošta et al. (2023)
*Entonaema cinnabarinum*	CNF 2/11047	Croatia		OQ863735	OQ864983	OQ877103	OQ877114	Pošta et al. (2023)
*Entonaema liquescens*	CNF 2/11263	USA		OQ869784	OQ865124	OQ877106	OQ877117	Pošta et al. (2023)
*Entonaema liquescens*	ENCB:RV_19274	Mexico		OR807997	OR807993	OR825474	OR825466	Reyes et al. (2024)
*Graphostroma platystomum*	CBS 270.87	France		JX658535	DQ836906	KY624296	HG934108	Zhang et al. (2006; LSU); Stadler et al. (2014; ITS); Koukol et al. (2015; *tub2*); Wendt et al. (2018; *rpb2*)
*Hypomontagnella barbarensis*	STMA 14081	Argentina	HT	MK131720	MK131718	MK135891	MK135893	Lambert et al. (2019)
*Hypomontagnella monticulosa*	MUCL 54604	French Guiana	ET	KY610404	KY610487	KY624305	KX271273	Wendt et al. (2018)
*Hypomontagnella submonticulosa*	CBS 115280	France		KC968923	KY610457	KY624226	KC977267	Kuhnert et al. (2014a; ITS, *tub2*); Wendt et al. (2018; LSU, *rpb2*)
*Hypomontagnella submonticulosa*	YMJ 351	France		JQ009316	N/A	N/A	AY951756	Hsieh et al. (2005)
*Hypomontagnella submonticulosa*	MUCL 57732	Iran		PX943592	PX943606	PX984355	PX984367	This study
*Hypomontagnella submonticulosa*	GUM 1613	Iran		PX943593	PX943607	PX984356	PX984368	This study
*Hypoxylon addis*	MUCL 52797	Ethiopia	HT	KC968931	ON954141	OP251037	KC977287	Kuhnert et al. (2014a; ITS, *tub2*); Cedeño-Sanchez et al. (2023b; LSU, *rpb2*)
*Hypoxylon aurantium*	MFLU 16-1202	Thailand	HT	MN047114	MN017878	N/A	N/A	Dayarathne et al. (2020)
*Hypoxylon aveirense*	MUM 19.40	Portugal	HT	MN053021	ON954142	OP251028	MN066636	Vicente et al. (2021; ITS, *tub2*); Cedeño-Sanchez et al. (2023b; LSU, *rpb2*)
*Hypoxylon baruense*	UCH 9545	Panama	HT	MN056428	ON954143	PP732079	MK908142	Cedeño–Sanchez et al. (2020; ITS, *tub2*); Cedeño-Sanchez et al. (2023b; LSU); Cedeño-Sanchez et al. (2025; *rpb2*)
*Hypoxylon canariense*	MUCL 47224	Spain	PT	ON792787	ON954140	OP251029	ON813073	Cedeño-Sanchez et al. (2023b)
*Hypoxylon carneum*	MUCL 54177	France		KY610400	KY610480	KY624297	KX271270	Wendt et al. (2018)
*Hypoxylon cercidicola*	CBS 119009	France		KC968908	KY610444	KY624254	KC977263	Kuhnert et al. (2014a; ITS, *tub2*); Wendt et al. (2018; LSU, *rpb2*)
*Hypoxylon chionostomum*	STMA 14060	Argentina		KU604563	ON954144	OP251030	ON813072	>Sir et al. (2016; ITS); Cedeño-Sanchez et al. (2023b; LSU, *rpb2*, *tub2*)
*Hypoxylon chrysalidosporum*	FCATAS 2710	China	HT	OL467294	OL615106	OL584222	OL584229	Ma et al. (2022)
*Hypoxylon cinnabarinum*	YMJ 8	Mexico		JN979408	N/A	N/A	AY951709	Hsieh et al. (2005)
*Hypoxylon cinnabarinum*	YMJ 43	Puerto Rico		JN979409	N/A	N/A	AY951708	Hsieh et al. (2005)
*Hypoxylon crocopeplum*	CBS 119004	France		KC968907	KY610445	KY624255	KC977268	Kuhnert et al. (2014a; ITS, *tub2*); Wendt et al. (2018; LSU, *rpb2*)
*Hypoxylon crocopeplum*	CNF 2/11316	Croatia		OQ865120	OQ869786	OQ877107	OQ877118	Pošta et al. (2023)
*Hypoxylon crocopeplum*	CNF 2/11317	Croatia		OQ865187	OQ869787	OQ877108	OQ877119	Pošta et al. (2023)
*Hypoxylon crocopeplum*	YMJ 7	Taiwan		JN979411	N/A	N/A	AY951711	Hsieh et al. (2005)
*Hypoxylon crocopeplum*	YMJ 11	USA		JN979410	N/A	N/A	AY951710	Hsieh et al. (2005)
*Hypoxylon crocopeplum*	ANM 1118	USA		JN673047	N/A	N/A	N/A	Raja et al. (2011)
*Hypoxylon crocopeplum*	GUM 1611	Iran		PX943594	PX944757	N/A	N/A	This study
*Hypoxylon cyclobalanopsidis*	FCATAS 2714	China	HT	OL467298	OL615108	OL584225	OL584232	Ma et al. (2022)
*Hypoxylon damuense*	FCATAS 4207	China	HT	ON075427	ON075433	ON093251	ON093245	Song et al. (2022a)
*Hypoxylon delonicis*	MFLU 16-1031	Thailand	HT	MT215503	MT386008	N/A	MT212215	Perera et al. (2020)
*Hypoxylon duranii*	ATCC 58730	Mexico	HT	PP718984	PP729636	PP732085	PP721316	Cedeño-Sanchez et al. (2025)
*Hypoxylon dussii*	MUCL 53766	Guadeloupe	HT	PP718981	PP729635	PP732081	PP721315	Cedeño-Sanchez et al. (2025)
*Hypoxylon erythrostroma*	MUCL 53759	Martinique		KC968910	ON954154	OP251031	KC977296	Kuhnert et al. (2014a; ITS); Cedeño-Sanchez et al. (2023b; LSU, *rpb2*)
*Hypoxylon erythrostroma*	YMJ 81	Mexico		JN979415	N/A	N/A	AY951715	Hsieh et al. (2005)
*Hypoxylon erythrostroma*	YMJ 90080602	Taiwan		JN979416	N/A	N/A	AY951716	Hsieh et al. (2005)
*Hypoxylon eurasiaticum*	MUCL 57720	Iran	HT	MW367851	N/A	MW373852	MW373861	Lambert et al. (2021)
*Hypoxylon fendleri*	MUCL 54792	French Guiana		KF234421	KY610481	KY624298	KF300547	Kuhnert et al. (2014a; ITS, *tub2*); Wendt et al. (2018; LSU, *rpb2*)
*Hypoxylon fendleri*	DSM 107927	USA		MK287533	MK287545	MK287558	MK287571	Sir et al. (2019)
*Hypoxylon fendleri*	DSM 107923	USA		N/A	MK287541	MK287554	MK287567	Sir et al. (2019)
*Hypoxylon fendleri*	YMJ HFA	Mexico		JN979417	N/A	N/A	AY951717	Hsieh et al. (2005)
*Hypoxylon fendleri*	YMJ 92092016	Taiwan		JN979418	N/A	N/A	AY951718	Hsieh et al. (2005)
*Hypoxylon ferrugineum*	CBS 141259	Austria		KX090079	N/A	N/A	KX090080	Friebes and Wendelin (2016)
*Hypoxylon fragiforme*	MUCL 51264	Germany	ET	KC477229	KM186295	MK887342	KX271282	Stadler et al. (2013; ITS); Daranagama et al. (2015; LSU); Sir et al. (2019; *rpb2*); Wendt et al. (2018; *tub2*)
*Hypoxylon fuscoides*	MUCL 52670	France	HT	ON792789	ON954145	OP251038	ON813076	Cedeño-Sanchez et al. (2023b)
*Hypoxylon fuscum*	CBS 113049	France	ET	KY610401	KY610482	KY624299	KX271271	Wendt et al. (2018)
*Hypoxylon gibriacense*	MUCL 52698	France	HT	KC968930	ON954146	OP251026	ON813074	Kuhnert et al. (2014a; ITS); Cedeño-Sanchez et al. (2023b; LSU, *rpb2*, *tub2*)
*Hypoxylon griseobrunneum*	CBS 331.73	India	HT	KY610402	KY610483	KY624300	KC977303	Kuhnert et al. (2014a; *tub2*); Wendt et al. (2018; ITS, LSU, *rpb2*)
*Hypoxylon guilanense*	MUCL 57726	Iran	HT	MT214997	MT214992	MT212235	MT212239	Pourmoghaddam et al. (2020)
*Hypoxylon haematostroma*	MUCL 53301	Martinique	ET	KC968911	KY610484	KY624301	KC977291	Kuhnert et al. (2014a; ITS, *tub2*); Wendt et al. (2018; LSU, *rpb2*)
*Hypoxylon hainanense*	FCATAS2712	China	HT	OL467296	OL616132	OL584224	OL584231	Ma et al. (2022)
*Hypoxylon hinnuleum*	MUCL 3621	USA	HT	MK287537	MK287549	MK287562	MK287575	Sir et al. (2019)
*Hypoxylon hongheensis*	HKAS 122663	China	HT	OM001336	OM001339	ON392009	ON468656	Yang et al. (2022)
*Hypoxylon howeanum*	MUCL 47599	Germany		AM749928	KY610448	KY624258	KC977277	Bitzer et al. (2008; ITS); Kuhnert et al. (2014a; *tub2*); Wendt et al. (2018; LSU, *rpb2*)
*Hypoxylon howeanum*	YMJ 388	France		JQ009323	N/A	N/A	AY951728	Hsieh et al. (2005)
*Hypoxylon howeanum*	UCH 9565	Panama		MN056427	N/A	N/A	MK908144	Cedeño–Sanchez et al. (2020)
*Hypoxylon howeanum*	MUCL 57730	Iran		PX943595	PX943608	PX984357	PX984369	This study
*Hypoxylon hypomiltum*	MUCL 51845	Guadeloupe		KY610403	KY610449	KY624302	KX271249	Wendt et al. (2018)
*Hypoxylon hyrcanense*	MUCL 57719	Iran	HT	PX943596	PX943609	PX984358	PX984370	This study
*Hypoxylon inaequale*	KUNCC 22-10798	China	HT	ON329812	ON329815	N/A	N/A	Jayawardena et al. (2022)
*Hypoxylon invadens*	MUCL 51475	France	HT	MT809133	MT809132	MT813037	MT813038	Becker et al. (2020)
*Hypoxylon investiens*	CBS 118183	Malaysia		KC968925	KY610450	KY624259	KC977270	Kuhnert et al. (2014a; ITS, *tub2*); Wendt et al. (2018; LSU, *rpb2*)
*Hypoxylon investiens*	CBS 118185 / STMA 05034	Ecuador		KC968924	KY610451	KY624260	KC977269	Kuhnert et al. (2014a; ITS, *tub2*); Wendt et al. (2018; LSU, *rpb2*)
*Hypoxylon investiens*	YMJ 89062905	Taiwan		JN979428	N/A	N/A	AY951730	Hsieh et al. (2005)
*Hypoxylon investiens*	MUCL 53307 / CBS 129034	Martinique		KC477239	N/A	N/A	KC977293	Kuhnert et al. (2014a)
*Hypoxylon investiens*	STMA 14058	Argentina		KU604568	N/A	N/A	KU159528	Sir et al. (2016)
*Hypoxylon investiens*	TBRC 16251	Thailand		OP856531	OP856521	OQ108848	OQ144968	Suetrong et al. (2023)
*Hypoxylon isabellinum*	MUCL 53308	Martinique	HT	KC968935	ON954155	OP251032	KC977295	Kuhnert et al. (2014a; ITS, *tub2*); Cedeño-Sanchez et al. (2023b; LSU, *rpb2*)
*Hypoxylon jecorinum*	YMJ 39	Mexico		JN979429	N/A	N/A	AY951731	Hsieh et al. (2005)
*Hypoxylon jianfengense*	FCATAS 845	China	HT	MW984546	MZ029707	MZ047260	MZ047264	Song et al. (2022b)
*Hypoxylon larissae*	FCATAS844	China	HT	MW984548	MZ029706	MZ047258	MZ047262	Song et al. (2022b)
*Hypoxylon larissae*	GUM 1602	Iran		PX943597	N/A	N/A	N/A	This study
*Hypoxylon larissae*	IRAN 4833C	Iran		PX943598	N/A	PX984359	PX984371	This study
*Hypoxylon larissae*	IRAN 4834C	Iran		PX943599	N/A	PX984360	PX984372	This study
*Hypoxylon laschii*	MUCL 52796	France		JX658525	ON954147	OP251027	ON813075	Stadler et al. (2014; ITS); Cedeño-Sanchez et al. (2023b; LSU, *rpb2*, *tub2*)
*Hypoxylon lateripigmentum*	MUCL 53304	Martinique	HT	KC968933	KY610486	KY624304	KC977290	Kuhnert et al. (2014a; ITS, *tub2*); Wendt et al. (2018; LSU, *rpb2*)
*Hypoxylon lateripigmentum*	MUCL 57716	Iran		PV522286	PV522295	PV524401	PV524410	Lambert et al. (2025)
*Hypoxylon lechatii*	MUCL 54609	French Guiana		KF923407	ON954148	OP251033	KF923405	Kuhnert et al. (2014b; ITS, *tub2*); Cedeño-Sanchez et al. (2023b; LSU, *rpb2*)
*Hypoxylon lenormandii*	CBS 119003	Ecuador		KC968943	KY610452	KY624261	KC977273	Kuhnert et al. (2014a; ITS, *tub2*); Wendt et al. (2018; LSU, *rpb2*)
*Hypoxylon lenormandii*	YMJ 92092009	Taiwan		JN979431	N/A	N/A	AY951733	Hsieh et al. (2005)
*Hypoxylon lienhwacheense*	MFLUCC 14-1231	Thailand		KU604558	MK287550	MK287563	KU159522	>Sir et al. (2016; ITS, *tub2*); Sir et al. (2019; LSU, *rpb2*)
*Hypoxylon lignicola*	MFLUCC 16-0926	China	HT	MK828609	MK835808	MN156534	N/A	Luo et al. (2019)
*Hypoxylon lividipigmentum*	BCRC 34077	Mexico	IT	JN979433	N/A	N/A	AY951735	Hsieh et al. (2005)
*Hypoxylon lividipigmentum*	STMA 14045	Argentina		ON792788	ON954149	PP732080	ON813077	Cedeño-Sanchez et al. (2023b; ITS, LSU, *tub2*); Cedeño-Sanchez et al. (2025; *rpb2*)
*Hypoxylon luteogranulatum*	BCC 79720	Thailand	HT	PP955304	PP955701	PP968827	PQ000351	Wongkanoun et al. (2025)
*Hypoxylon macrocarpum*	CBS 119012	Germany		ON792785	ON954151	OP251034	ON813071	Cedeño-Sanchez et al. (2023b)
*Hypoxylon mangrovei*	MFLU 18-0559	Thailand	HT	MN047116	MN017880	N/A	MN077053	Dayarathne et al. (2020)
*Hypoxylon medogense*	FCATAS 4061	China	HT	ON075425	ON075431	ON093249	ON093243	Song et al. (2022a)
*Hypoxylon munkii*	MUCL 53315	Martinique		KC968912	ON954153	OP251035	KC977294	Kuhnert et al. (2014a; ITS, *tub2*); Cedeño-Sanchez et al. (2023b; LSU, *rpb2*)
*Hypoxylon musceum*	MUCL 53765	Guadeloupe		KC968926	KY610488	KY624306	KC977280	Kuhnert et al. (2014a; ITS, *tub2*); Wendt et al. (2018; LSU, *rpb2*)
*Hypoxylon ochraceum*	MUCL 54625	Martinique	ET	KC968937	N/A	KY624271	KC977300	Kuhnert et al. (2014a; ITS, *tub2*); Wendt et al. (2018; *rpb2*)
*Hypoxylon olivaceopigmentum*	DSM 107924	USA	HT	MK287530	MK287542	MK287555	MK287568	Sir et al. (2019)
*Hypoxylon perforatum*	STMA 23134	USA	ET	PP718982	PP729634	PP732084	PP721314	Cedeño-Sanchez et al. (2025)
*Hypoxylon perforatum*	CBS 115281	France		KY610391	KY610455	KY624224	KX271250	Wendt et al. (2018)
*Hypoxylon perforatum*	DSM 107930	USA		MK287529	MK287540	MK287553	MK287566	Sir et al. (2019)
*Hypoxylon perforatum*	MUCL 57728	Iran		PX943600	PX943610	PX984361	PX984373	This study
*Hypoxylon petriniae*	CBS 114746	France	HT	KY610405	KY610491	KY624279	KX271274	Wendt et al. (2018)
*Hypoxylon phuphaphetense*	TBRC 16277	Thailand	HT	OP856538	OP856528	OQ108849	OQ144973	Preedanon et al. (2023)
*Hypoxylon pilgerianum*	STMA 13455	Martinique		KY610412	KY610412	KY624308	KY624315	Wendt et al. (2018)
*Hypoxylon polyporoideum*	YMJ 15	Taiwan		JQ009311	N/A	N/A	AY951747	Hsieh et al. (2005)
*Hypoxylon polyporoideum*	YMJ 56	Thailand		JQ009312	N/A	N/A	AY951748	Hsieh et al. (2005)
*Hypoxylon porphyreum*	CBS 119022	France		KC968921	KY610456	KY624225	KC977264	Kuhnert et al. (2014a; ITS, *tub2*); Wendt et al. (2018; LSU, *rpb2*)
*Hypoxylon pseudofuscum*	DSM 112038	Germany	HT	MW367857	MW367848	MW373858	MW373867	Lambert et al. (2021)
*Hypoxylon pseudoinvestiens*	MUCL 57729	Iran	HT	PX943601	PX943611	PX984362	PX984374	This study
*Hypoxylon pseudoinvestiens*	GUM 1605	Iran		PX943602	PX943612	PX984363	PX984375	This study
*Hypoxylon pulicicidum*	CBS 122622	Martinique	HT	JX183075	KY610492	KY624280	JX183072	Bills et al. (2012; ITS, *tub2*); Wendt et al. (2018; LSU, *rpb2*)
*Hypoxylon rickii*	MUCL 53309	Martinique	ET	KC968932	KY610416	KY624281	KC977288	Kuhnert et al. (2014a; ITS, *tub2*); Wendt et al. (2018; LSU, *rpb2*)
*Hypoxylon rubiginosum*	MUCL 52887	Germany	ET	KC477232	KY610469	KY624266	KY624311	Stadler et al. (2013; ITS); Wendt et al. (2018; LSU, *rpb2*, *tub2*)
*Hypoxylon rutilum*	YMJ 181	France		N/A	N/A	N/A	AY951752	Hsieh et al. (2005)
*Hypoxylon samuelsii*	MUCL 51843	Guadeloupe	ET	KC968916	KY610466	KY624269	KC977286	Kuhnert et al. (2014a; ITS, *tub2*); Wendt et al. (2018; LSU, *rpb2*)
*Hypoxylon sofainense*	MUCL 54170	Guadeloupe	HT	PP718983	PP729633	PP732083	PP721313	Cedeño-Sanchez et al. (2025)
*Hypoxylon sporistriatatunicum*	UCH 9542	Panama	HT	MN056426	ON954150	OP251036	MK908140	Cedeño-Sanchez et al. (2020; ITS, *tub2*); Cedeño-Sanchez et al. (2023b; LSU, *rpb2*)
*Hypoxylon subgilvum*	STMA 24034	Panama		PP718985	PP729637	PP732078	PP721317	Cedeño-Sanchez et al. (2025)
*Hypoxylon subgilvum*	YMJ 246	USA		JQ009314	N/A	N/A	AY951754	Hsieh et al. (2005)
*Hypoxylon subgilvum*	YMJ 88113007	Taiwan		JQ009315	N/A	N/A	AY951755	Hsieh et al. (2005)
*Hypoxylon subticinense*	MUCL 53752	French Guiana		KC968913	ON954152	PP732082	KC977297	Kuhnert et al. (2014b; ITS, *tub2*); Cedeño-Sanchez et al. (2023b; LSU); Cedeño-Sanchez et al. (2025; *rpb2*)
*Hypoxylon teeravasati*	NFCCI-4216	India	HT	KY863509	MF385274	MG986895	MG986894	Phookamsak et al. (2019)
*Hypoxylon texense*	DSM 107933	USA	HT	MK287536	MK287548	MK287561	MK287574	Sir et al. (2019)
*Hypoxylon ticinense*	CBS 115271	France		JQ009317	KY610471	KY624272	AY951757	Hsieh et al. (2005; ITS, *tub2*); Wendt et al. (2018; LSU, *rpb2*)
*Hypoxylon ticinense*	MUCL 47714	Germany		KY610410	KY610470	N/A	KX271259	Wendt et al. (2018)
*Hypoxylon ticinense*	GUM 1606	Iran		PX943603	PX943613	PX984364	PX984376	This study
*Hypoxylon ticinense*	MUCL 57731	Iran		PX943604	PX943614	PX984365	PX984377	This study
*Hypoxylon trugodes*	MUCL 54794	Sri Lanka	ET	KF234422	KY610493	KY624282	KF300548	Kuhnert et al. (2014a; ITS, *tub2*); Wendt et al. (2018; LSU, *rpb2*)
*Hypoxylon vogesiacum*	CBS 115273	France		KC968920	KY610417	KY624283	KX271275	Kuhnert et al. (2014a; ITS); Wendt et al.(2018; LSU, *rpb2*, *tub2*)

*Hypoxylon wuzhishanense*	FCATAS 2708	China	HT	OL467292	OL615104	OL584220	OL584227	Ma et al. (2022)
*Hypoxylon xmatkuilense*	UADY:PR_3	Mexico	HT	OR807999	OR807990	OR825476	OR825467	Reyes et al. (2024)
*Hypoxylon zangii*	FCATAS 4029	China	HT	ON075423	ON075429	ON093247	ON093241	Song et al. (2022b)
*Hypoxylon zhaotongensis*	GMB CC1168	China	HT	OP597690	OP598100	N/A	N/A	Zhang et al. (2023)
*Jackrogersella cohaerens*	CBS 119126	Germany		KY610396	KY610497	KY624270	KY624314	Wendt et al. (2018)
*Jackrogersella minutella*	CBS 119015	Portugal		KY610381	KY610424	KY624235	KX271240	Kuhnert et al. (2017; *tub2*); Wendt et al. (2018; ITS, LSU, *rpb2*)
*Jackrogersella multiformis*	CBS 119016	Germany	ET	KC477234	KY610473	KY624290	KX271262	Stadler et al. (2013; ITS); Kuhnert et al. (2017; *tub2*); Wendt et al. (2018; LSU, *rpb2*)
*Kretzschmaria deusta*	MUCL 57705	Iran		MH084755	OP359327	OP359596	OP359601	Pourmoghaddam et al. (2018; ITS); Pourmoghaddam et al. (2022a; LSU, *rpb2*, *tub2*);
*Kretzschmaria hedjaroudei*	MUCL 57706	Iran	HT	MH084757	OP359328	OP359597	OP359602	Pourmoghaddam et al. (2018; ITS); Pourmoghaddam et al. (2022a; LSU, *rpb2*, *tub2*);
*Nemania hyrcana*	MUCL 57704	Iran	HT	OP359332	OP359329	OP359598	OP359603	Pourmoghaddam et al. (2022b)
*Nemania serpens*	MUCL 57702	Iran		OP359334	OP359331	OP359600	OP359605	Pourmoghaddam et al. (2022b)
*Parahypoxylon papillatum*	ATCC 58729	USA	HT	KC968919	KY610454	KY624223	KC977258	Kuhnert et al. (2014a; ITS, *tub2*); Wendt et al. (2018; LSU, *rpb2*)
*Parahypoxylon ruwenzoriense*	MUCL 51392	D. R. Congo	HT	ON792786	ON954156	OP251039	ON813078	Cedeño-Sanchez et al. (2023b)
*Phylacia globosa*	STMA 18042	Argentina		OQ437889	OQ437885	OQ453168	OQ453172	Lambert et al. (2023)
*Phylacia lobulata*	STMA 18032	Argentina	HT	OQ437892	OQ437882	OQ453166	N/A	Lambert et al. (2023)
*Phylacia surinamensis*	STMA 18044	Argentina		OQ437891	OQ437887	OQ453167	N/A	Lambert et al. (2023)
*Pyrenopolyporus bambusicola*	BCC89355	Thailand	HT	OP304856	OP304876	OP981624	OQ101839	Wongkanoun et al. (2023)
*Pyrenopolyporus cinereopigmentosus*	BCC89382	Thailand	HT	OP304860	OP304882	OP981627	OQ101843	Wongkanoun et al. (2023)
*Pyrenopolyporus hunteri*	MUCL 52673	Ivory Coast	ET	KY610421	KY610472	KY624309	KU159530	Kuhnert et al. (2017; *tub2*); Wendt et al. (2018; ITS, LSU, *rpb2*)
*Pyrenopolyporus laminosus*	MUCL 53305	Martinique	HT	KC968934	KY610485	KY624303	KC977292	Kuhnert et al. (2014a; ITS, *tub2*); Wendt et al. (2018; LSU, *rpb2*)
*Pyrenopolyporus macrosporus*	BCC89373	Thailand	HT	OP304870	OP304879	OP981621	OQ101844	Wongkanoun et al. (2023)
*Pyrenopolyporus nicaraguensis*	CBS 117739	Burkina Faso		AM749922	KY610489	KY624307	KC977272	Bitzer et al. (2008; ITS); Kuhnert et al. (2014a; *tub2*); Wendt et al. (2018; LSU, *rpb2*)
*Rhopalostroma angolense*	CBS 126414	Ivory Coast		KY610420	KY610459	KY624228	KX271277	Wendt et al. (2018)
*Ruwenzoria pseudoannulata*	MUCL 51394	D. R. Congo	HT	KY610406	KY610494	KY624286	KX271278	Wendt et al. (2018)
*Thamnomyces dendroideus*	CBS 123578	French Guiana	HT	FN428831	KY610467	KY624232	KY624313	Stadler et al. (2010; ITS); Wendt et al. (2018; LSU, *rpb2*, *tub2*)
*Xylaria arbuscula*	CBS 126415	Germany		KY610394	KY610463	KY624287	KX271257	Wendt et al. (2018)
*Xylaria hypoxylon*	CBS 122620	Sweden	ET	KY610407	KY610495	KY624231	KX271279	Wendt et al. (2018)

Maximum parsimony (MP) analyses were performed with PAUP v. 4.0a169 (Swofford 2002). All molecular characters were unordered and given equal weight; analyses were performed with gaps treated as missing data; the COLLAPSE command was set to MINBRLEN. MP analysis of the combined multilocus matrix was conducted using 1,000 replicates of heuristic search with random addition of sequences and subsequent TBR branch swapping (MULTREES option in effect, steepest descent option not in effect). Bootstrap analyses with 1,000 replicates were performed in the same way but using 10 rounds of random sequence addition and subsequent branch swapping during each bootstrap replicate.

Maximum likelihood (ML) analyses were performed with RAxML as implemented in raxmlGUI v. 2.0 (Edler et al. 2021) using the ML + rapid bootstrap setting with 1,000 bootstrap replicates (Felsenstein 1985) and the GTRGAMMA substitution model. In the Results and Discussion, bootstrap values ≤ 70% are considered low, between 70% and 90% intermediate, and ≥ 90% high. Phylogenetic trees were visualized in PAUP v. 4.0a169 (Swofford 2002) and finalized with Adobe Illustrator® v. CC 2025 (Adobe Inc., San Jose, California, USA).

### HPLC profiling

Stromata were extracted essentially as described in Kuhnert et al. (2017). Briefly, stromatal tissue was scraped with a spatula and collected in an Eppendorf tube, covered with acetone, centrifuged, and 60–100 µL of extract was collected for subsequent HPLC-DAD-UV/Vis-ESI-MS analysis. System parameters and settings were identical to those described by Kuhnert et al. (2014b). The resulting spectra were analyzed using DataAnalysis V4.4 (Bruker Daltonics) and compared with internal databases for identification of discernible compound peaks and stromata-fingerprints.

## Results

### Molecular phylogeny

**Figure 1.**
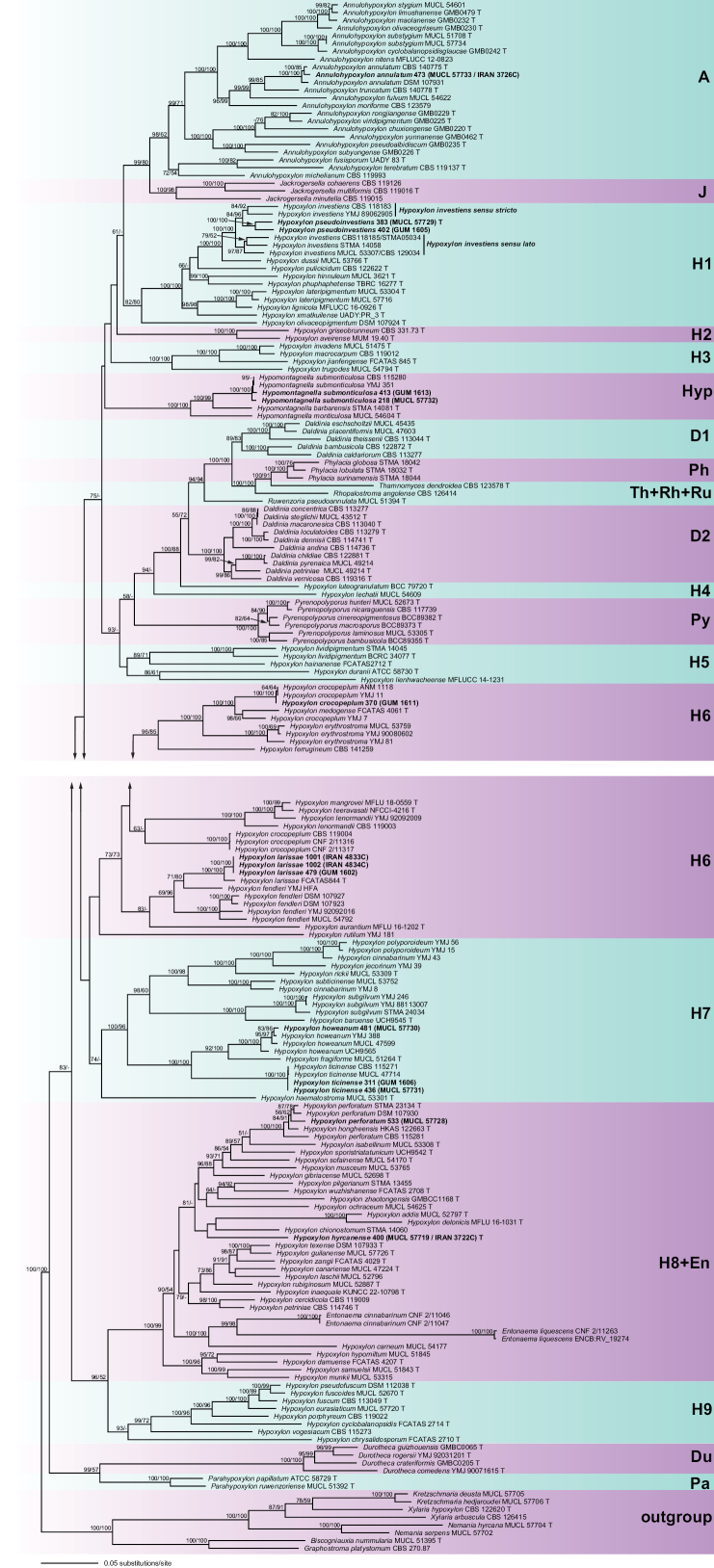
Phylogram of the best ML tree (lnL = –130,791.8061) revealed by RAxML from an analysis of the combined ITS–LSU–*rpb2*–*tub2* matrix of selected Hypoxylaceae. Strains in bold were sequenced in the current study. ML and MP BS support values above 50% are given at the first and second positions, respectively, above or below the branches. “T” indicates type material.

Of the 4,247 characters of the combined matrix, 1,831 were parsimony-informative (342 in ITS, 237 in LSU, 531 in *rpb2*, and 721 in *tub2*). The phylogram of the best ML tree (lnL = –130,791.8061) obtained by RAxML is shown in Fig. 1. Maximum parsimony analyses resulted in five most parsimonious trees (tree length = 29,758; Consistency Index [CI] = 0.140; Retention Index [RI] = 0.606; Rescaled Consistency Index [RC] = 0.085; and Homoplasy Index [HI] = 0.860) (not shown). Estimated base frequencies were as follows: A = 0.239064, C = 0.268281, G = 0.256547, and T = 0.236106, with substitution rates AC = 1.200848, AG = 4.610122, AT = 1.267940, CG = 0.951380, CT = 6.220641, and GT = 1.000000. Except for some deeper nodes of the backbone and a few minor topological differences within terminal clades, tree topologies of the MP trees were compatible with the ML tree shown in Fig. 1.

The phylogenies revealed a paraphyly of *Hypoxylon*, with the genera *Annulohypoxylon*, *Daldinia*, *Durotheca*, *Entonaema*, *Jackrogersella*, *Hypomontagnella*, *Parahypoxylon*, *Phylacia*, *Pyrenopolyporus*, *Rhopalostroma*, *Ruwenzoria*, and *Thamnomyces* embedded within the former, as reported in previous studies (e.g., Cedeño-Sanchez et al. 2023b; Lambert et al. 2025). All of the latter genera appeared monophyletic except for *Daldinia* (Fig. 1).

Isolates from the present study were placed into six clades in the phylogenetic tree (Fig. 1). In subclade A, the Iranian isolate of *Annulohypoxylon annulatum* (MUCL 57733/IRAN 3726C) clustered together with the ex-epitype culture (CBS 140775) with maximum support, their sequences being almost identical.

In subclade H1, the new species *H. pseudoinvestiens* (MUCL 57729/402/383) clustered as a sister species to *Hypoxylon investienssensu stricto* (CBS 118183 from Malaysia and YMJ 89062905 from Taiwan) with high ML and MP BS support. These two groups clustered as sister to *H. investienssensu lato* (MUCL 53307 from Martinique, STMA 14058 from Argentina, and CBS 118185 from Ecuador) with maximum ML and MP BS support.

In subclade Hyp, the Iranian isolates (MUCL 57732/218/413) clustered with *Hypomontagnella submonticulosa* (CBS 115280 and YMJ 351, both from France) with maximum ML and MP BS support.

In subclade H6, the Iranian isolate (GUM 1611) clustered together with *Hypoxylon crocopeplum* (YMJ 11 and ANM 1118, both from the USA) with maximum ML and MP BS support. These three isolates clustered as sister to *H. crocopeplum* (YMJ 7 from Taiwan) and *Hypoxylon medogense* (FCATAS 4061 from China) with maximum ML and MP BS support and were separate from European *H. crocopeplum* (CBS 119004 from France and CNF 2/11316 and CNF 2/11317 from Croatia). Moreover, the Iranian isolates (IRAN 4833C/IRAN 4834C/479) clustered together with *Hypoxylon larissae* (FCATAS844 from China) with maximum ML and MP BS support.

In subclade H7, the Iranian isolate (MUCL 57730/481) clustered together with *Hypoxylon howeanum* (MUCL 47599 from Germany, YMJ 388 from France, and UCH 9565 from Panama) with maximum ML and MP BS support. In addition, the Iranian isolates (MUCL 57731/311/436) clustered together with *Hypoxylon ticinense* (CBS 115271 from France and MUCL 47714 from Germany) with maximum ML and MP BS support.

In subclade H8+En, the position of the strain (MUCL 57719/IRAN 3722C/400H) did not receive bootstrap support but was resolved in a separate lineage showing a higher nucleotide difference compared to other closely related species, which supports its independence. Furthermore, the Iranian isolate (MUCL 57730/533) resolved in a cluster formed by *Hypoxylon perforatum* STMA 23134 (ex-epitype culture from the USA), DSM 107930 (from the USA), and CBS 115281 (from France), amidst *Hypoxylon hongheensis* (HKAS 122663 from China), receiving maximum ML and MP BS support. This suggests a potential synonymy of *H. hongheensis* with *H. perforatum* (Cedeño-Sanchez et al. 2025). Unfortunately, the Chinese taxon was never studied by chemotaxonomic methods, and no type studies were performed in the respective study by Yang et al. (2022), which only used morphological data from the literature and sequence data deposited in GenBank for comparison with their own collections. This practice is unfortunately not unparalleled and has probably already led to the erection of several unnecessary later synonyms.

The comparison of the multi-gene matrix tree (ITS–LSU–*rpb2*–*tub2*) with the *tub2* single-locus tree revealed no significant topological conflicts, and the phylogenetic signals of both trees were compatible (see Suppl. material 1).

### Taxonomic part

#### Annulohypoxylon annulatum

Taxon classificationFungiXylarialesHypoxylaceae

(Schwein.) Y.M. Ju, J.D. Rogers & H.M. Hsieh, Mycologia 97(4): 857. 2005

0ECB9B68-2551-5FAC-871D-64609EB7A616

Fig. 2

##### Teleomorph.

Stromata superficial, hemispherical, up to 2 cm long × 0.2–0.8 cm wide, with inconspicuous perithecial mounds; surface Olivaceous (48) to brown when young, becoming Brown Vinaceous (84); blackish granules immediately beneath surface; with KOH-extractable pigments Yellowish Green (18) or Green (20). Perithecia spherical to obovoid 0.5–0.75 mm high × 0.25–0.65 mm wide; ostioles coarsely papillate, encircled with a convex truncatum-type disc 0.3–0.5 mm diam. Asci 8-spored, cylindrical, with weakly amyloid, discoid apical apparatus, 0.5 µm high × 1–1.5 µm wide, stipe up to 50 µm, and spore-bearing portion 60–70 × 4–5 µm. Ascospores brown to dark brown, unicellular, ellipsoid-inequilateral, with narrowly rounded ends, (7–)8.0–11.0(–12) × 3–4 µm, with straight germ slit spore-length on the convex side; perispore dehiscent in 10% KOH, smooth with a thickening on the convex side; epispore smooth.

**Figure 2.**
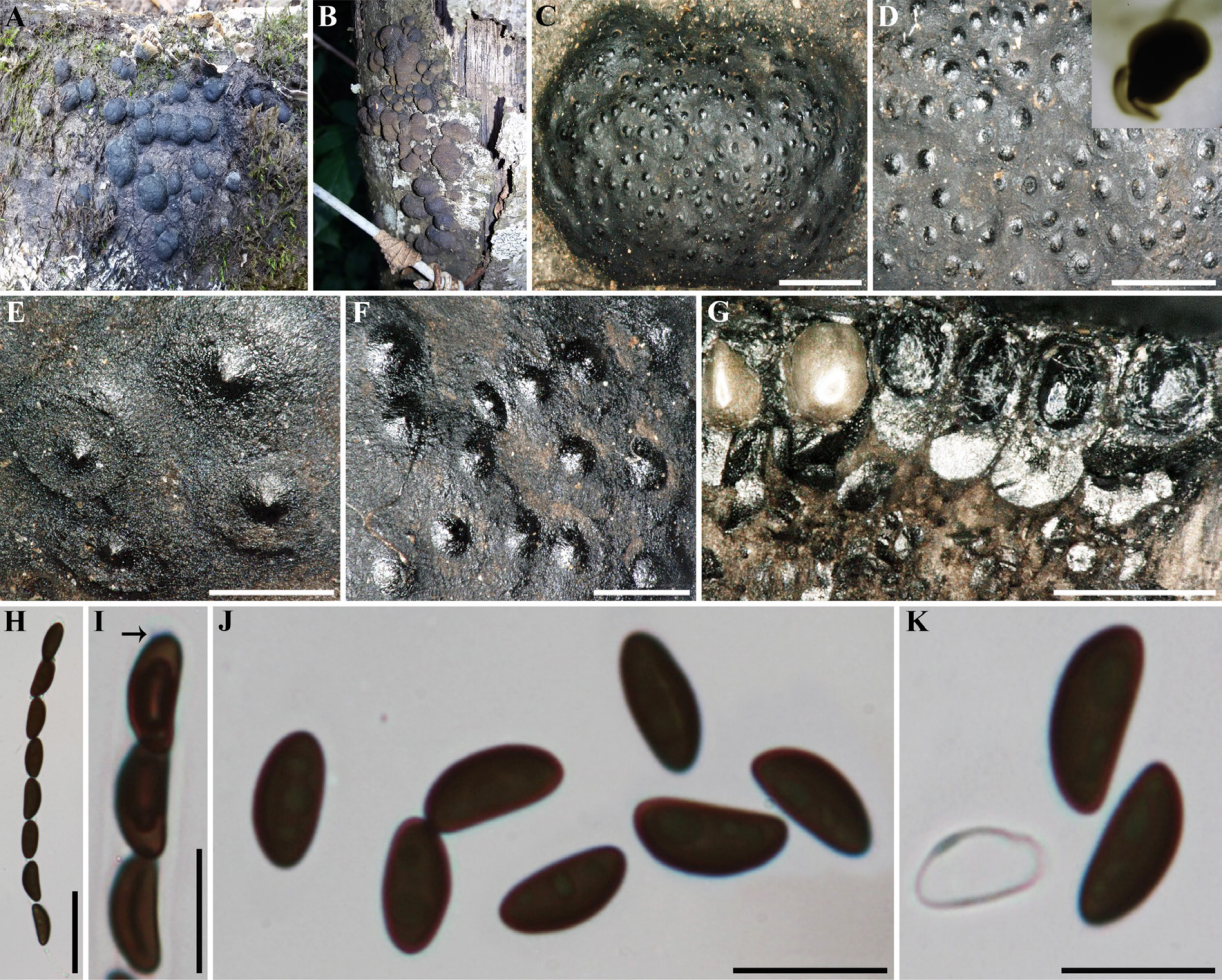
*Annulohypoxylon annulatum* (GUM 1614). **A, B**. Old and young stromatal habit; **C**. Close-up view of stroma surface; **D**. Close-up view of stroma surface with KOH-extractable pigments from stroma; **E, F**. Close-up view of ostiolar discs; **G**. Stroma in vertical section showing perithecia; **H**. Mature ascus in water; **I**. Ascus tip in Melzer’s reagent (black arrow); **J**. Ascospore in water; **K**. Ascospores in 10% KOH with dehiscent perispore showing thickening. Scale bars: 2 mm (**C**); 1.5 mm (**D**); 0.5 mm (**E, F**); 1 mm (**G**); 20 µm (**H**); 10 µm (**I–K**).

##### Secondary metabolites.

Not observed.

##### Specimens examined.

Iran • Guilan Province, Rasht County, Saravan forest, 37°04'26"N, 49°38'13"E, 183 m elev., on fallen branch of *Quercus castaneifolia*, 9 October 2016, M.J. Pourmoghaddam (GUM 1614; living culture MUCL 57733; IRAN 3726C); • Guilan Province, Siahkal County, Ziaratgah forest, 37°08'07.34"N, 49°55'36.14"E, 260 m elev., on dead branches (host unknown), 14 October 2016, M.J. Pourmoghaddam.

##### Notes.

*Annulohypoxylon annulatum* and *A. truncatum* share stromatic features, such as truncatum-type ostiolar discs and KOH-extractable pigments. *Annulohypoxylon annulatum* can be differentiated from *A. truncatum* by the shape of its stromata (hemispherical or massively pulvinate in *A. annulatum* and hemispherical, glomerate, pulvinate, or effused-pulvinate in *A. truncatum*), stromatal secondary metabolites (hypoxylonols C and F in *A. annulatum* and truncatone A and truncaquinones A and B in *A. truncatum*), and molecular phylogeny (Kuhnert et al. 2017; Li et al. 2025). Most of the characters of the Iranian specimens are in accordance with the type description (Ju and Rogers 1996; Kuhnert et al. 2017), aside from insignificant variations in the size of ascospores [(7–)8.0–11.0(–12) × 3–4 vs. 7.5–10.5(–11) × 3.5–5(–6) µm].

#### Hypomontagnella submonticulosa

Taxon classificationFungiXylarialesHypoxylaceae

(Y.M. Ju & J.D. Rogers) Sir, L. Wendt & C. Lamb., 2019

276E4CB0-DCD4-5DF9-A5B7-C7D695AD92D3

Fig. 4

##### Teleomorph.

Stromata superficial, pulvinate to effused-pulvinate, up to 5 cm long × 0.4–2.5 cm wide, with inconspicuous perithecial mounds; surface Rust (39) to Bay (6) in young stromata, Brown Vinaceous (84) to blackish in mature stromata, shiny; blackish woody to carbonaceous tissue immediately beneath surface and between perithecia, KOH-extractable pigments pale Livid Violet (79) in young stromata, without apparent KOH-extractable pigments in mature stromata. Perithecia obovoid to spherical, 0.3–0.4 mm high × 0.25–0.35 mm wide. Ostioles higher than the stromatal surface, minutely conical papillate, surrounded by a black disc 100–152 μm diam. Asci 8-spored, cylindrical, with amyloid, discoid apical apparatus, 1–2 µm high × 2.5–3 µm wide, stipe up to 50 µm long, and spore-bearing portion 70–85 × 6–10 µm. Ascospores brown, unicellular, ellipsoid-inequilateral, with broadly to less frequently narrowly rounded ends, 10–13(–14) × 4–5.5 µm, with straight germ slit less than spore-length on convex side; perispore indehiscent in 10% KOH.

**Figure 3.**
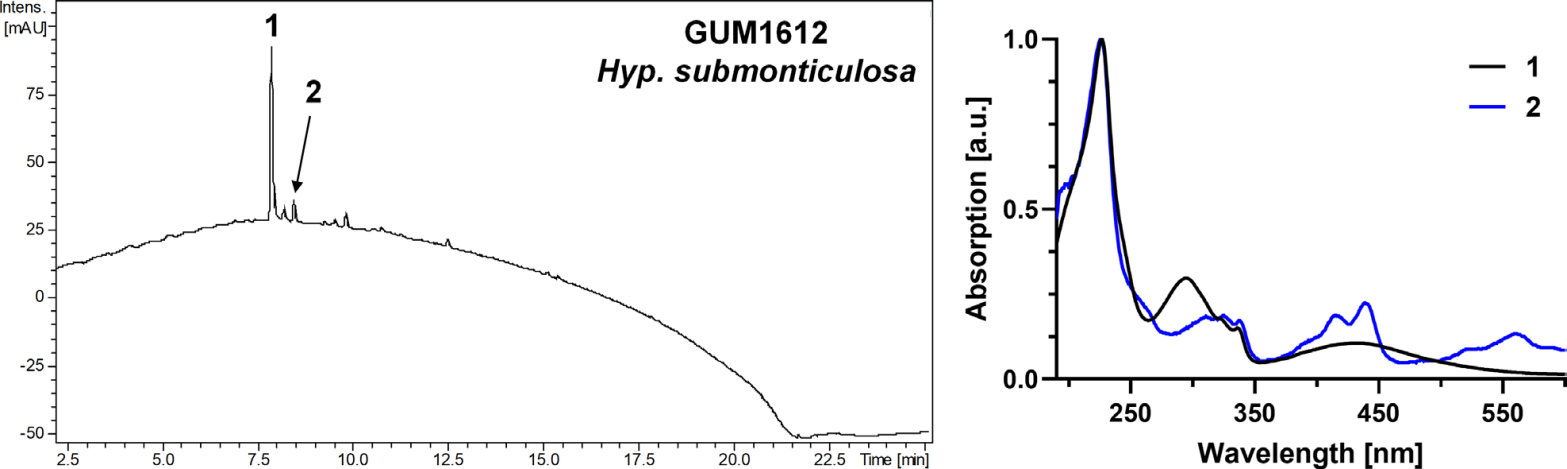
Left: HPLC-UV chromatogram (210 nm) of a stromatal acetone extract of GUM 1612. Right: UV-Vis spectrum of designated peaks. The stromatal extract contains a compound resembling hinnulin A (1) and traces of BNT (2).

**Figure 4.**
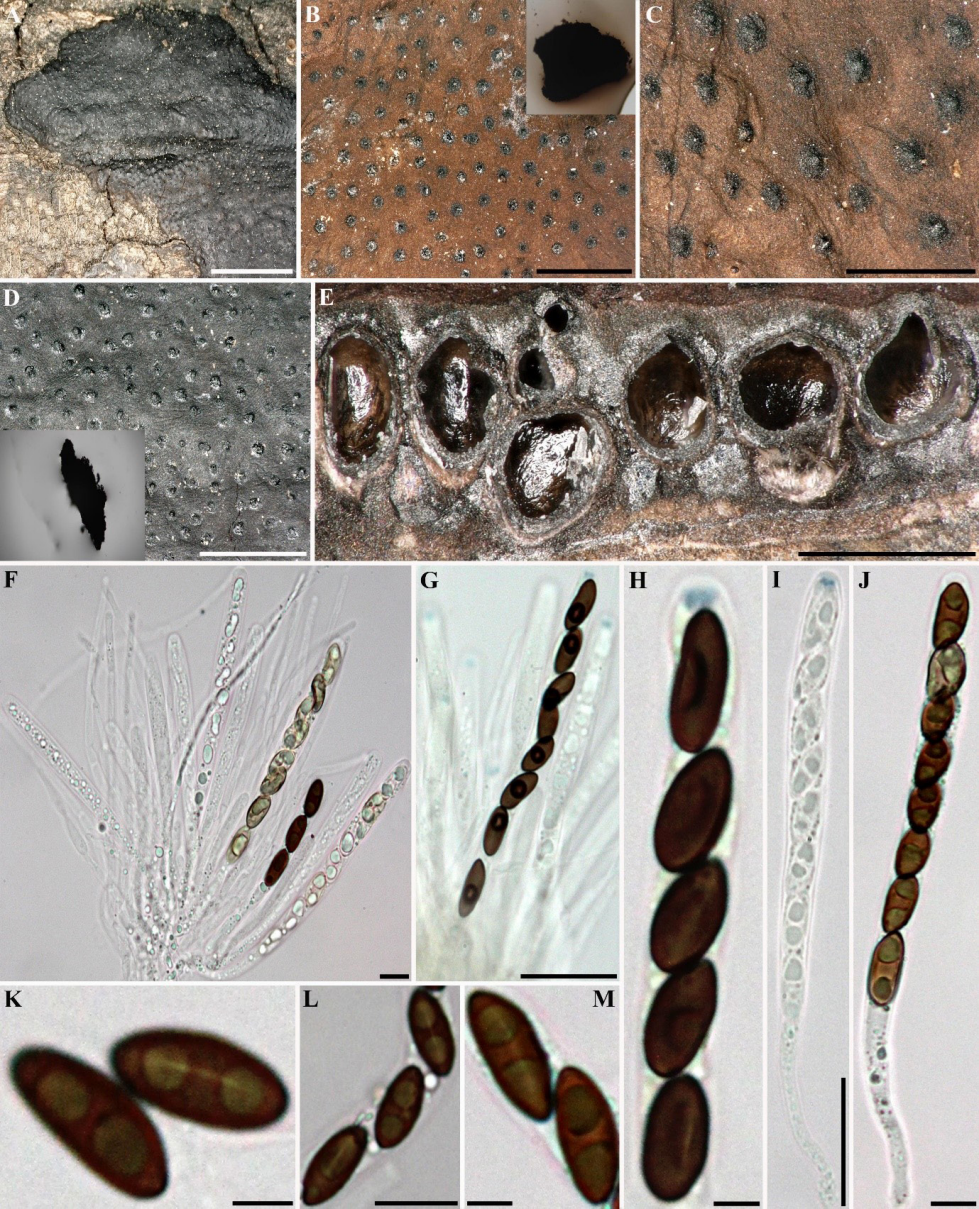
*Hypomontagnella submonticulosa* (GUM 1612). **A**. Stromatal habit; **B**. Close-up view of stromatal surface with KOH-extractable pigments in young stromata; **C**. Stromatal surface showing papillate ostiolar disc; **D**. Close-up view of stromatal surface without KOH-extractable pigments in mature stromata; **E**. Stroma in vertical section showing perithecia; **F**. Immature and mature asci and paraphyses in water; **G**. Immature and mature asci in Melzer’s reagent; **H, I**. Ascus in Melzer’s reagent; **J**. Ascus in water; **K–M**. Ascospores in water, with straight germ slit less than spore-length. Scale bars: 5 mm (**A**); 1 mm (**B**); 0.5 mm (**C–E**); 20 µm (**F–G**); 10 µm (**H, K–M**).

##### Cultures and anamorph.

Colonies on OA covering a 9 cm Petri dish in 2 weeks, at first whitish, becoming Pale Luteous (11) with sporulation, velvety, azonate to faintly zonate, with diffuse margins. Conidiogenous structure branching virgariella-like as defined by Ju and Rogers (1996). Conidiophores hyaline, smooth to finely roughened. Conidiogenous cell hyaline, smooth, 16–30 × 1.5–2 µm. Conidia hyaline, smooth to finely roughened, ellipsoid, 4–5.5 × 2.5–3.5 µm.

##### Secondary metabolites.

Traces of the binaphthyls hinnulin A (1) and BNT (2) in young stroma of GUM 1612 (see Fig. 3). No detectable secondary metabolites in GUM 1613.

##### Specimens examined.

Iran • Guilan Province, Shaft County, Babarekab forest, 37°00'27"N, 49°20'23"E, 289 m elev., on dead branches (host unknown), 15 September 2016, M.J. Pourmoghaddam (GUM 1612; living culture MUCL 57732); • Guilan Province, Talesh County, Gisoom forest, 37°39'41"N, 49°00'31"E, 500 m elev., on fallen branch of *Quercus castaneifolia*, 20 October 2016, M.J. Pourmoghaddam (GUM 1613).

##### Notes.

This specimen from our collection shares characters with the type specimen (Ju and Rogers 1996; Lambert et al. 2019), aside from insignificant variations in the size of ascospores [10–13(–14) × 4–5.5 vs. 9–13.5 × 4–5.8 µm]. *Hypomontagnella submonticulosa* was previously accommodated in *Hypoxylon* and recently segregated into a new genus (Lambert et al. 2019). It can be differentiated from *Hypomontagnella barbarensis* by its much smaller ascospores [10–13(–14) × 4–5.5 vs. 13–19.3 × 6.9–9.4 µm] and germ slit (straight to slightly oblique germ slit less than spore-length vs. straight, spore-length).

#### Hypoxylon crocopeplum

Taxon classificationFungiXylarialesHypoxylaceae

Berk. & M.A. Curtis, Grevillea 4(no. 30): 49. 1875

6754AF5A-66B1-5812-85FB-816A34268EA0

Fig. 6

##### Teleomorph.

Stromata superficial, effused-pulvinate, up to 8 cm long × 0.4–2 cm wide, with conspicuous perithecial mounds; surface Orange (7), Sienna (8), Apricot (42) in young stromata, Bay (6) to Sepia (63) in mature stromata; orange red granules immediately beneath surface and between perithecia, with KOH-extractable pigments Orange (7). Perithecia obovoid to slightly tubular, 0.2–0.5 mm high × 0.2–0.4 mm wide. Ostioles lower than or at the same level as the stromatal surface. Asci 8-spored, cylindrical, with discoid apical apparatus lightly bluing or bluing in Melzer’s iodine reagent, 0.5–1 µm high × 2–2.5 µm wide, stipe up to 100 µm, and spore-bearing portion 70–96 × 7–9 µm. Ascospores smooth, unicellular, brown to dark brown, ellipsoid-inequilateral, with narrowly rounded ends, 11–15(–15.8) × 5–6.8 µm, with straight to slightly sigmoid germ slit spore-length on convex side; perispore dehiscent in 10% KOH, inconspicuously to conspicuously striated; epispore smooth.

##### Secondary metabolites

**Figure 5.**
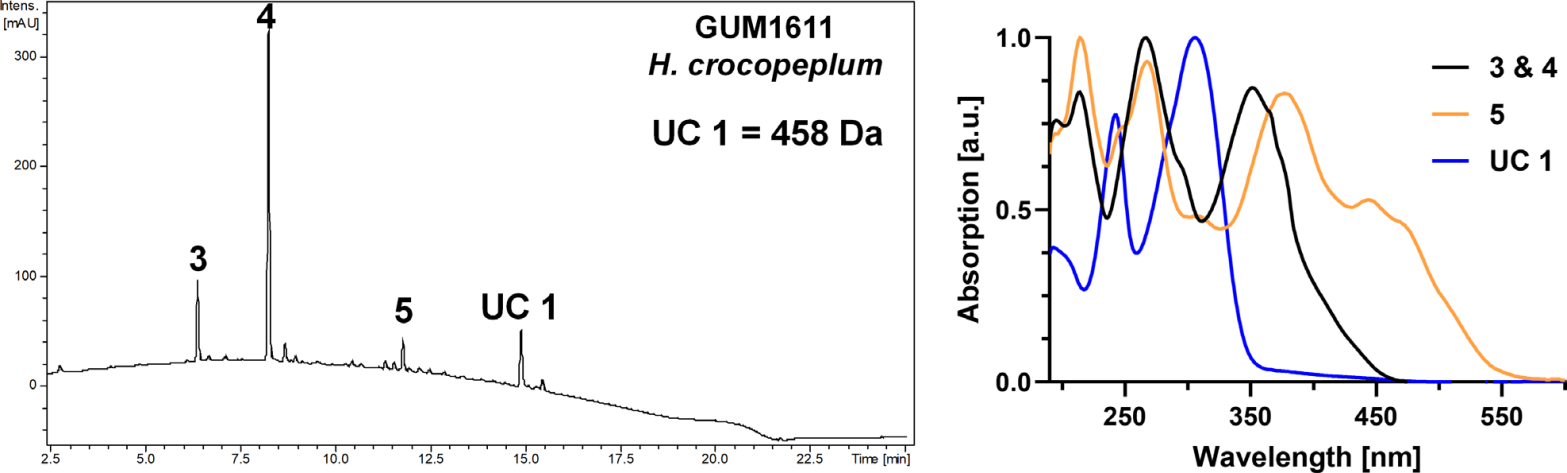
Left: HPLC-UV chromatogram (210 nm) of a stromatal acetone extract of GUM 1611. Right: UV-Vis spectra of designated peaks, revealing mitrobrinol (3), mitorubrinol acetate (4), rutilin D (5), and an unidentified compound (UC 1).

**(see Fig. 5)**. Mitorubrinol (3), mitorubrinol acetate (4), rutilin D (5) and an unidentified compound with a mass of 458 Da (UC 1).

**Figure 6.**
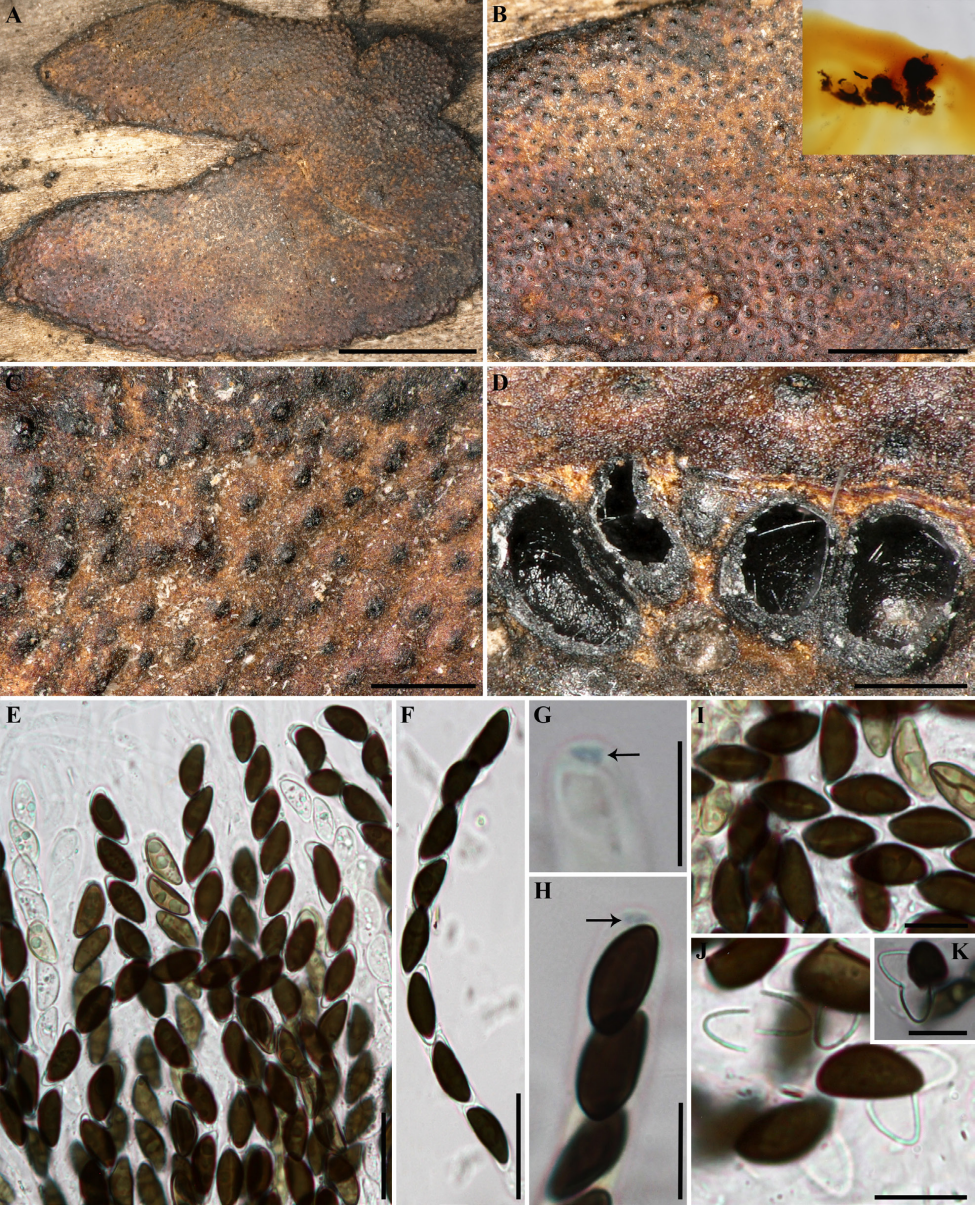
*Hypoxylon crocopeplum* (GUM 1611). **A**. Stromatal habit; **B**. Close-up view of stromatal surface, with stromatal pigments in 10% KOH; **C**. Section of stroma showing conspicuous perithecial mounds; **D**. Stroma in horizontal section showing perithecia, granules beneath the surface and between perithecia and ostioles; **E**. Immature and mature asci in 10% KOH; **F**. Ascus in water; **G**. Immature ascus tip in Melzer’s reagent; **H**. Mature ascus tip in Melzer’s reagent; **I**. Ascospores in 10% KOH with straight to slightly sigmoid germ slit spore-length on convex side; **J**. Ascospores in 10% KOH with dehiscent perispore; **K**. Detail of striated perispore. Scale bars: 5 mm (**A**); 2 mm (**B**); 0.5 mm (**C**); 0.25 mm (**D**); 20 µm (**E, F**); 10 µm (**G–K**).

##### Specimen examined.

USA • South Carolina, on corticated wood of oak?, as *Sphaeria crocopepla*, H. W. Ravenel, Car. Inf. 1906 Berk. & M. A. Curtis, ined. (K, holotype); Iran • Guilan Province, Chaboksar County, Sarvelat forest, 36°57'23"N, 50°32'56"E, 590 m elev., on dead branches (host unknown), 23 September 2016, M.J. Pourmoghaddam (GUM 1611).

##### Notes.

With thin, effused, dull orange-brown stromata, orange-yellow stromatal pigments, and dense orange KOH-extractable pigments, combined with dark brown inequilateral ascospores with a straight germ slit, this *Hypoxylon* specimen keys out to *H. crocopeplum* or *H. subgilvum*. *H. crocopeplum* differs from the latter by the ostioles being lower than or at the same level as the stromatal surface vs. ostioles umbilicate and inconspicuous, the size of ascospores [(13.4–)13.8–17.1(–17.5) × (7.1–)7.3–8.3(–8.6) vs. (8.9–)9.1–9.9(–10.6) × (4.2–)4.5–5.0(–5.1) μm], and the anamorphic structure [a virgariella-like conidiogenous structure was observed in culture on YMG (unpublished data) and on JF-03233, vs. on the natural substrate with a virgariella- to nodulisporium-like conidiogenous structure, in culture on OA nodulisporium-like] (Fournier et al. 2015).

The salient microscopic features of the European *H. crocopeplum* are the rather broad ascospores (11.4–14.4 × 6–7.2 μm) with narrowly rounded ends and their conspicuously sigmoid germ slit. Such a sigmoid germ slit is known only from *H. fuscum* among European species of *Hypoxylon* (http://pyrenomycetes.free.fr/hypoxylon/html/Hypoxylon_crocopeplum.htm).

*Hypoxylon crocopeplum* was originally described from the southeastern USA and is widespread in the tropics and subtropics and highly variable in the size of ascospores and in the shape of perithecia (Ju and Rogers 1996). Based on molecular data, the species concept of *H. crocopeplum* defined by Ju and Rogers (1996) was restricted by Hsieh et al. (2005) by accommodating specimens with thick stromata and long tubular perithecia in *H. polyporoideum*. However, the wide ascospore size range of *H. crocopeplum* remained unchanged, making it difficult to discriminate the two species without data on the anamorph or without molecular data (Fournier et al. 2015). We have found that the material from France and Iran has the same HPLC profile as the old type specimen from the USA housed in Kew, with rubiginosin-like azaphilones being prevalent.

Our phylogenetic analysis showed that *H. crocopeplum* is currently polyphyletic, as specimens morphologically identified as *H. crocopeplum* phylogenetically fall into three separate clades (see Fig. 1). Given the lack of further phenotypic evidence, this issue is currently unresolvable. Fresh North American material needs to be studied in detail and compared to a significant number of materials from other geographic areas. Moreover, it would be desirable to select an epitype that can be cultured and studied for its anamorphic morphology and molecular data.

#### Hypoxylon howeanum

Taxon classificationFungiXylarialesHypoxylaceae

Peck, Ann. Rep. N.Y. St. Mus. 24: 98. 1872

621B23A0-D8D2-51A0-8536-134971E1C71E

##### Teleomorph.

Stromata superficial, hemispherical to spherical, sessile, up to 0.5 cm long × 0.1–0.4 cm wide, with inconspicuous to conspicuous perithecial mounds; surface Fulvous (43), Rust (39), or Dark Brick (60); orange red granules immediately beneath surface, with KOH-extractable pigments Orange (7) to Rust (39); white granules between perithecia. Perithecia spherical to obovoid, 0.3–0.5 mm high × 0.15–0.35 mm wide. Ostioles lower than or at the same level as the stromatal surface. Asci 8-spored, cylindrical, with amyloid, discoid apical apparatus, 0.5–1 µm high × 1.5–2 µm wide, stipe up to 70 µm long, and spore-bearing portion 50–65 × 4.5–6 µm. Ascospores brown to dark brown, unicellular, ellipsoid-inequilateral, with narrowly rounded ends, 6.5–8 × 3–4 µm, with straight to slight sigmoid germ slit spore-length on convex side; perispore dehiscent in 10% KOH, smooth; epispore smooth.

##### Secondary metabolites

**Figure 7.**
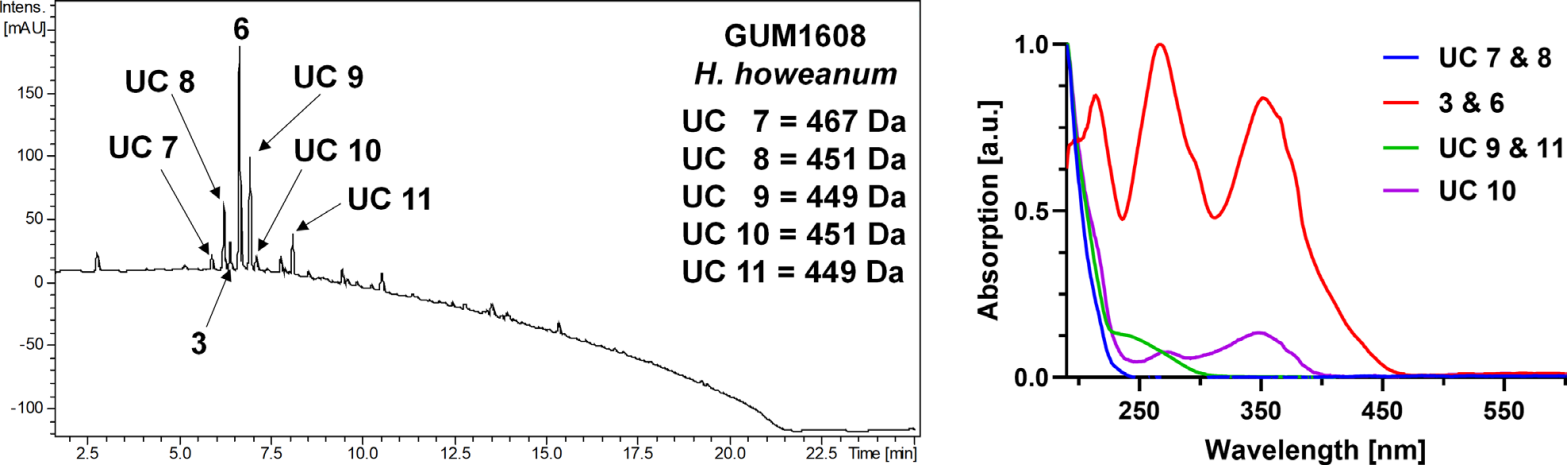
Left: HPLC-UV chromatogram (210 nm) of a stromatal acetone extract of GUM 1608. Right: UV-Vis spectrum of designated peaks. Stromatal extracts contained mitorubrinol and mitorubrinic acid (3, 6), as well as several unidentifiable compounds resembling cytochalasins (UC 7–11; see the structure of fragiformin A, 13, for an example).

**(see Fig. 7)**. Contains mitorubrinol (3), mitorubrinic acid (6) and several unidentified compounds resembling cytochalasins (UC 7–11).

##### Specimens examined.

Iran • Guilan Province, Siahkal County, Ziaratgah forest, 37°08'07.34"N, 49°55'36.14"E, 260 m elev., on fallen branch of *Parrotia persica*, 14 October 2016, M.J. Pourmoghaddam (GUM 1608; living culture MUCL 57730); • Guilan Province, Siahkal County, Ziaratgah forest, 37°08'07.34"N, 49°55'36.14"E, 260 m elev., on fallen branch of *Parrotia persica*, 14 October 2016, M.J. Pourmoghaddam (GUM 1609); • Guilan Province, Astaneh-ye Ashrafiyeh County, SafraBasteh forest, 37°20'19"N, 49°58'26"E, 14 m elev., on fallen branch of *Quercus castaneifolia*, 3 October 2016, M.J. Pourmoghaddam.

##### Notes.

*Hypoxylon howeanum* is morphologically and phylogenetically close to *H. fragiforme* and commonly shares stromatal secondary metabolites of the mitorubrin type, but not the rubiginosin-type family (Stadler and Fournier 2006). They are the only two species characterized by having two colors of granules: orange-red granules immediately beneath the stromatal surface and white granules between perithecia (Ju and Rogers 1996). *Hypoxylon howeanum* differs from the latter in having smaller ascospores [6.5–8 × 3–4 vs. (10.5–)11–15 × 5–6.5(–7) µm] and by a broader geographical and host range. Most of the features of the Iranian specimens are in accordance with previous descriptions (Ju and Rogers 1996); however, the ascospores were slightly smaller [6.5–8 × 3–4 vs. 7–9.5(–10) × 3–4.5 µm].

#### Hypoxylon hyrcanense

Taxon classificationFungiXylarialesHypoxylaceae

Pourmoghaddam sp. nov.

191119F2-FF36-525D-A039-6CE994A4C4E0

862211

Fig. 9

##### Holotype.

Iran • Guilan Province, Siahkal County, 37°57'17"N, 48°52'16"E, 1160 m elev., on fallen wood of *Quercus castaneifolia*, 3 October 2016, M.J. Pourmoghaddam, (GUM 1601; living culture MUCL 57719).

##### Etymology.

The epithet is derived from “Hyrcania,” an ancient biogeographical region, located in the south of the Caspian Sea where the specimen was collected.

##### Teleomorph.

Stromata superficial, effused-pulvinate, up to 3 cm long × 0.5–1.5 cm wide, with conspicuous perithecial mounds; surface Red (2) to Brick (59); Scarlet (5) to Orange (7) granules beneath the surface and between the perithecia, with KOH-extractable pigments Luteous (12). Perithecia obovoid to cylindrical, 0.15–0.2 mm high × 0.1–0.16 mm wide. Ostioles umbilicate, inconspicuous. Asci 8-spored, cylindrical, with amyloid, discoid apical apparatus, 0.5–1 µm high × 1.5–2.5 µm wide, stipe up to 200 µm, and spore-bearing portion 58–80 × 4.5–6.5 µm. Ascospores smooth, unicellular, brown to dark brown, ellipsoid, inequilateral with narrowly rounded ends, 13–15 × 5.5–6.5 µm, with straight germ slit spore-length on convex side; perispore dehiscent in 10% KOH, conspicuous coil-like ornamentation in SEM; epispore smooth.

##### Cultures and anamorph.

Colonies on OA covering a 9 cm Petri dish in 4 weeks, at first white, becoming pale Luteous (11) and Smoke Gray (105), felty, azonate with diffuse margins, finally becoming Isabelline (65) to Honey (64). Conidiophores and conidiogenous cells in culture not observed. Conidia hyaline, smooth, ellipsoid to cylindrical, 5–6 × 2–3 µm.

##### Secondary metabolites

**Figure 8.**
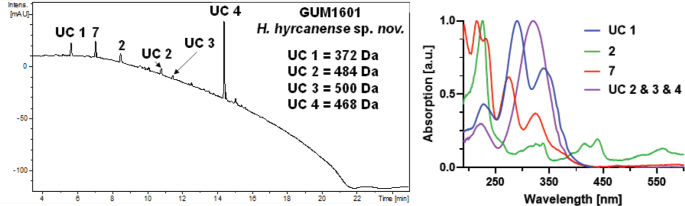
Left: HPLC-UV chromatogram (210 nm) of a stromatal acetone extract of GUM 1601. Right: UV-Vis spectrum of designated peaks. The stromatal extract contains BNT (2), a compound resembling daldinal B (7), and unknown secondary metabolites (UC 1–4). Mass numbers are given in Da to facilitate assignment in future studies.

**(see Fig. 8)**. BNT (2), a compound resembling daldinal B (7) and unknown secondary metabolites (UC 1–UC 4).

**Figure 9.**
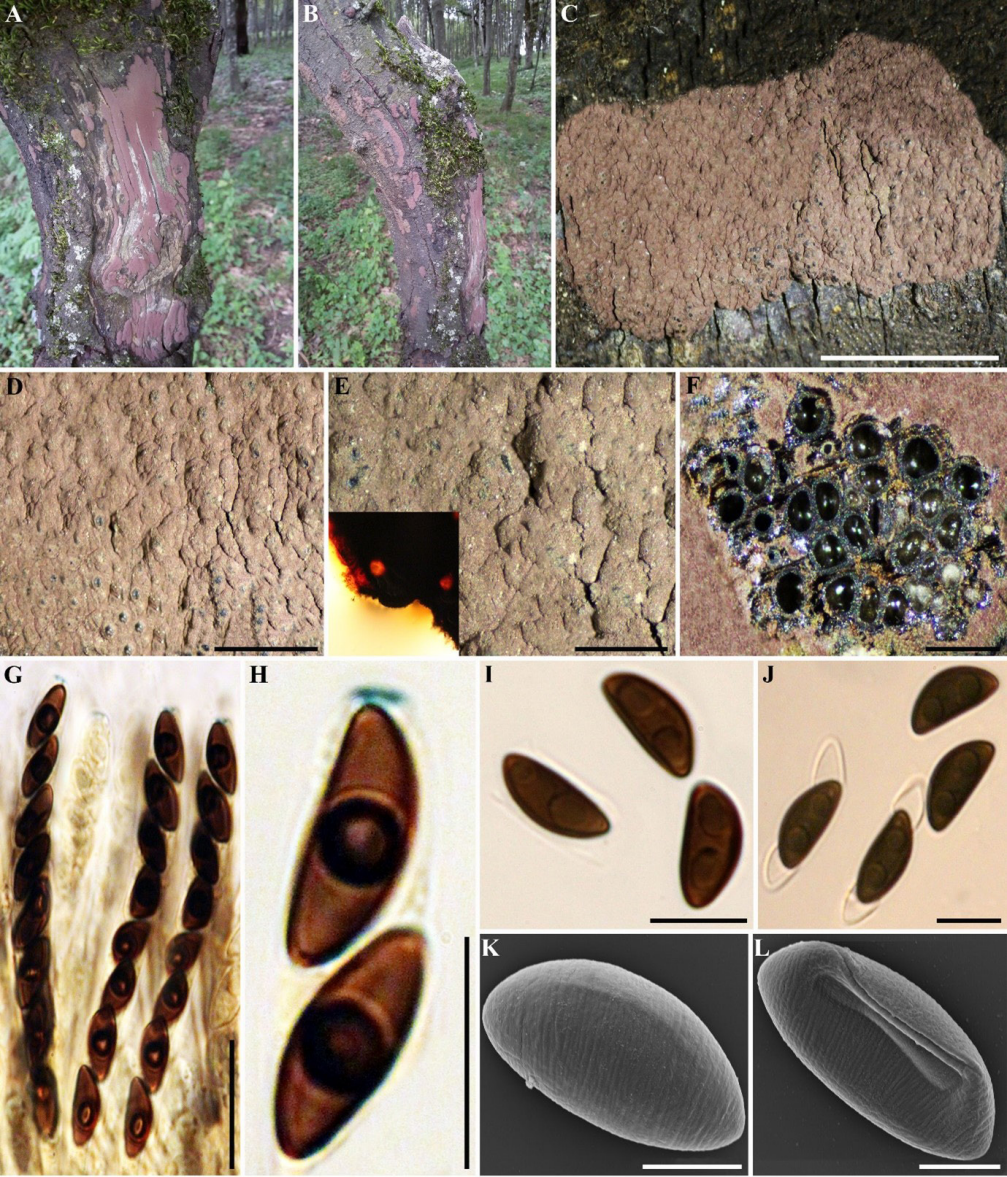
*Hypoxylon hyrcanense* (holotype GUM 1601). **A–C**. Stromatal habit; **D, E**. Close-up view of stromatal surface, with stromatal pigments in 10% KOH; **F**. Stroma in horizontal section showing perithecia and granules; **G**. Immature and mature asci in Melzer’s reagent; **H**. Ascus tip in Melzer’s reagent; **I**. Ascospores in water; **J**. Ascospores in 10% KOH with dehiscent perispore; **K, L**. Ascospore under SEM. Scale bars: 5 mm (**C**); 1.25 mm (**D**); 0.5 mm (**E**); 0.25 mm (**F**); 20 µm (**G**); 10 µm (**H–J**); 4 µm (**K**); 3 µm (**L**).

##### Notes.

*Hypoxylon ochraceum* and *H. chionostomum* are phylogenetically close to this new taxon, but both species can be easily differentiated from the new species. *Hypoxylon ochraceum* can be differentiated by its different stromatal shape (glomerate, frequently confluent), larger ascospores [12–16(–18) × 5.5–7.5(–8) vs. 13–15 × 5–6.5 µm], with a straight germ slit less than spore-length, and an indehiscent perispore in KOH. Moreover, it lacks mitorubrin-type derivatives present in *H. ochraceum* (Kuhnert et al. 2014a). *Hypoxylon chionostomum* can be differentiated by its major BNT (2), stromatal shape (glomerate), larger perithecia (1–1.4 mm), and larger ascospores (25–31 × 14–18 µm). In addition, this fungus seems to be endemic to the Neotropics (Sir et al. 2016).

#### Hypoxylon larissae

Taxon classificationFungiXylarialesHypoxylaceae

Hai X. Ma & Z. K. Song, Phytotaxa 538(3): 219 (2022)

2033A628-BD6D-571A-893A-126C91008378

Figs 11, 12

##### Teleomorph.

Stromata superficial, effused-pulvinate to pulvinate, up to 6 cm long × 0.3–2 cm wide, with conspicuous perithecial mounds; surface Scarlet (5) to Rust (39); Orange (7) granules beneath the surface and between the perithecia, with KOH-extractable pigments Orange (7) to Scarlet (5). Perithecia cylindrical to obovoid, 0.25–0.45 mm high × 0.22–0.33 mm wide. Ostioles umbilicate, inconspicuous. Asci 8-spored, cylindrical, with amyloid, discoid apical apparatus, 1–1.5 µm high × 2–3 µm wide, stipe up to 80 µm, and spore-bearing portion 80–100 × 9–12 µm. Ascospores smooth, unicellular, brown to dark brown, ellipsoid, inequilateral with narrowly rounded ends, 14–18 × 5.5–8.5 µm, with straight germ slit spore-length on convex side; perispore dehiscent in 10% KOH; epispore smooth.

##### Cultures and anamorph.

Colonies on OA covering a 9 cm Petri dish in 2 weeks, at first white, becoming Pale Luteous (11), felty, azonate with diffuse margins, finally becoming Umber (9). Conidiogenous structure approaching a virgariella-like branching pattern as defined by Ju and Rogers (1996), (Fig. 12C–G). Conidiophores hyaline, smooth to finely roughened. Up to three conidiogenous cells at the end of each terminus; hyaline, smooth to finely roughened, 14–26 × 2–3 µm. Conidia hyaline, smooth, ellipsoid to obovoid, 4–5 × 2.5–3 µm.

##### Secondary metabolites

**Figure 10.**
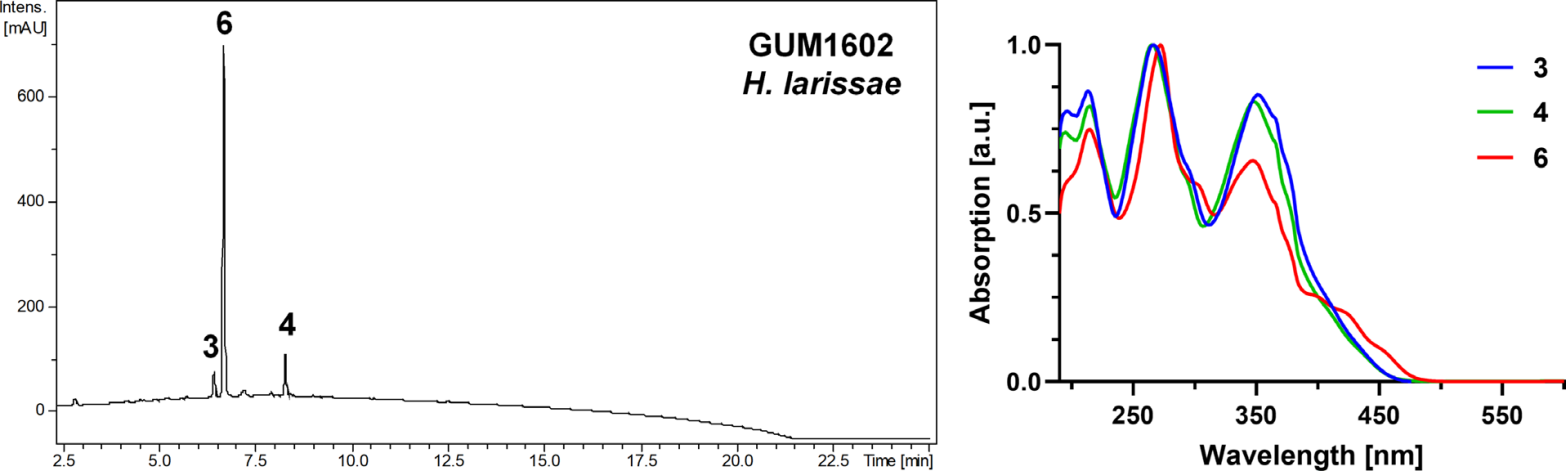
Left: HPLC-UV chromatogram (210 nm) of a stromatal acetone extract of GUM 1602. Right: UV-Vis spectrum of designated peaks. The stromatal extract contains mitorubrinol, mitorubrinol acetate, and mitorubrinic acid (3–4, 6).

**(see Fig. 10)**. Mitorubrinol (3), mitorubrinol acetate (4), and mitorubrinic acid (6).

**Figure 11.**
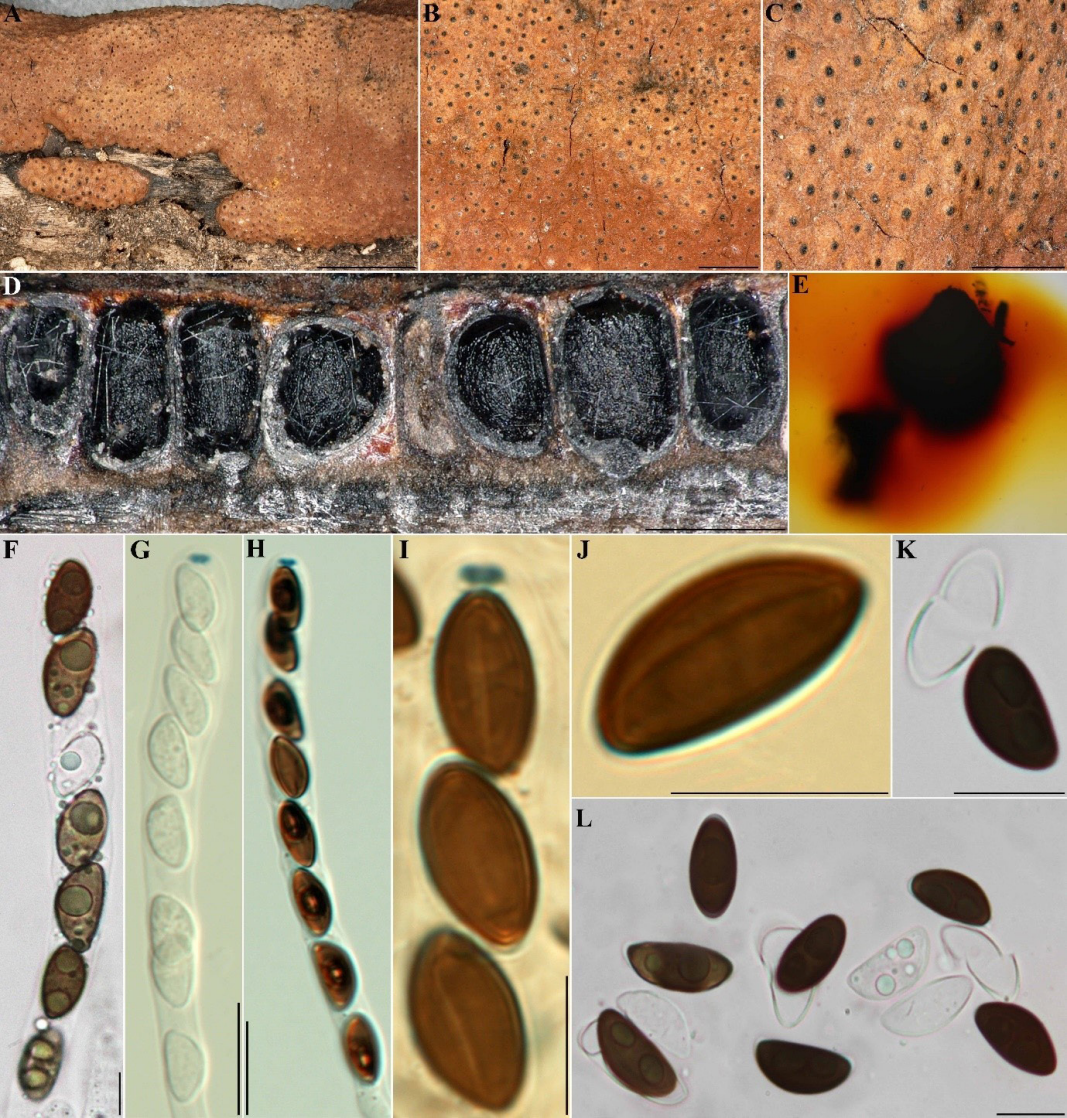
*Hypoxylon larissae* (GUM 1602). **A**. Stromatal habit; **B, C**. Close-up view of stromatal surface; **D**. Stroma in vertical section showing perithecia; **E**. KOH-extractable pigments from stroma; **F**. Immature ascus in water; **G**. Immature ascus in Melzer’s reagent; **H**. Mature ascus in Melzer’s reagent; **I**. Ascus tip in Melzer’s reagent; **J**. Ascospore in Melzer’s reagent with straight germ slit; **K, L**. Ascospores in 10% KOH with dehiscent perispore. Scale bars: 4 mm (**A**); 1 mm (**B, C**); 0.5 mm (**D**); 20 µm (**F–H**); 10 µm (**I–L**).

**Figure 12.**
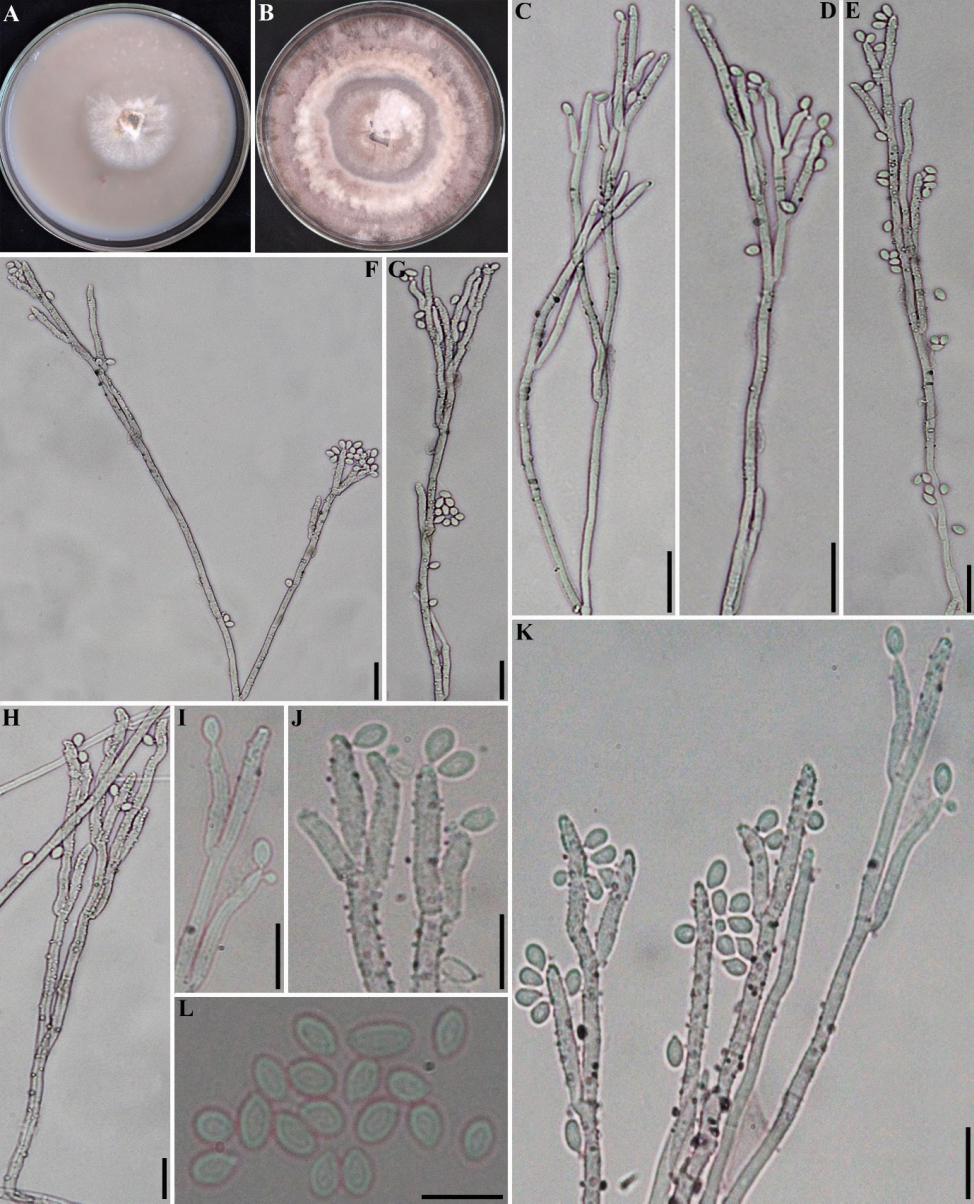
Culture and anamorphic structures of *Hypoxylon larissae* (IRAN 4833C) on OA. **A, B**. Surface of colony after 1 and 10 weeks of incubation (left to right); **C–H, K**. General view of anamorph structure with virgariella-like branching patterns; **I, J**. Conidiogenous cells with immature conidia; **L**. Mature conidia. Scale bars: 20 µm (**C–H**); 10 µm (**I–L**).

##### Specimens examined.

Iran • Rasht County, Saravan forest, 37°04'26"N, 49°38'13"E, 183 m elev., on fallen branch of *Quercus castaneifolia*, 12 October 2021, M.J. Pourmoghaddam (GUM 1648; living culture IRAN 4833C); • Guilan Province, Rasht County, Saravan forest, 37°04'26"N, 49°38'13"E, 183 m elev., on fallen branch of *Quercus castaneifolia*, 11 November 2021, M.J. Pourmoghaddam (GUM 1649; living culture IRAN 4834C); • Guilan Province, Rasht County, Saravan forest, 37°04'26"N, 49°38'13"E, 183 m elev., on fallen branch of *Quercus castaneifolia*, 9 October 2016, M.J. Pourmoghaddam (GUM 1602); Mazandaran Province, Chalous County, Saravan forest, 36°38'15"N, 51°24'47"E, 344 m elev., on fallen branch of *Quercus castaneifolia*, 30 October 2017, M.J. Pourmoghaddam (GUM 1650).

##### Notes.

Most of the characters of the Iranian specimens are in accordance with the type specimen (Song et al. 2022a), aside from the size of ascospores [14–18 × 5.5–8.5 vs. 15.5–23(–23.6) × 7.3–10.6 µm]. The anamorphic structure of *H. larissae* was not described by Song et al. (2022a). In the present study, we report for the first time a virgariella-like conidiogenous structure in culture for this species. *Hypoxylon fendleri* is phylogenetically close to *H. larissae* but differs by its smaller ascospores [(8–)9–12 × 4–5.5 µm] and a sigmoid germ slit spore-length on the convex side. In addition, it was originally described from Venezuela and is commonly found throughout the tropics (Ju and Rogers 1996; Sir et al. 2019). The HPLC analyses showed no detectable differences between reports for *H. fendleri* collected in the USA and *H. larissae* collected in Iran.

#### Hypoxylon perforatum

Taxon classificationFungiXylarialesHypoxylaceae

(Schwein.) Fr., Summa veg. Scand., Section Post. (Stockholm): 384. 1849

BBCD56C2-61C0-5D13-BC52-02B65DD3C063

Fig. 14

##### Teleomorph.

Stromata superficial, pulvinate to effused-pulvinate, up to 12 cm long × 0.1–0.5 cm wide, with inconspicuous to conspicuous perithecial mounds; surface Brown Vinaceous (84); dark brown granules immediately beneath surface and between perithecia, with KOH-extractable pigments Amber (47) to Citrine (13). Perithecia spherical, 0.2–0.35 mm high × 0.2–0.3 mm wide. Ostioles lower than the stromatal surface, mainly covered with white substances. Asci 8-spored, cylindrical, with amyloid, discoid apical apparatus, 0.5–1.5 µm high × 1.5–2.5 µm wide, stipe up to 60 µm long and spore-bearing portion 60–80 × 5–8.5 µm. Ascospores brown to dark brown, unicellular, ellipsoid-inequilateral, with narrowly rounded ends, 10–13 × 4–5.5 µm, with straight to slight sigmoid germ slit spore-length on convex side; perispore dehiscent in 10% KOH, smooth; epispore smooth.

##### Secondary metabolites

**Figure 13.**
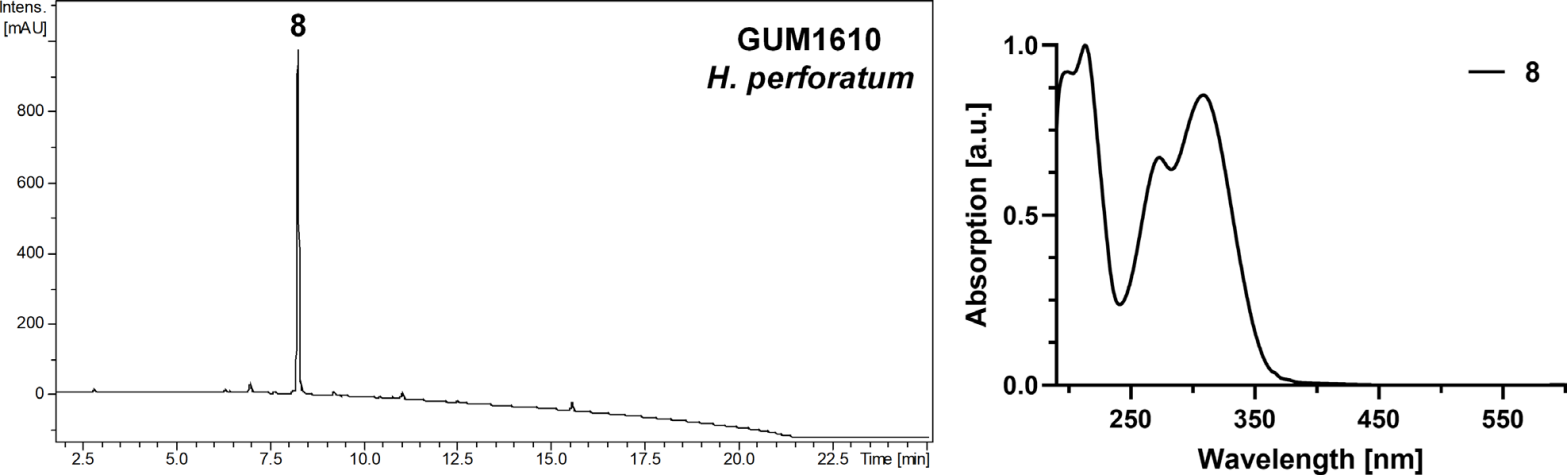
Left: HPLC-UV chromatogram (210 nm) of a stromatal acetone extract of GUM 1610. Right: UV-Vis spectrum of designated peaks. The stromatal extract contains hypomiltin as the major metabolite (8).

**(see Fig. 13)**. Contains hypomiltin as major metabolite (8).

**Figure 14.**
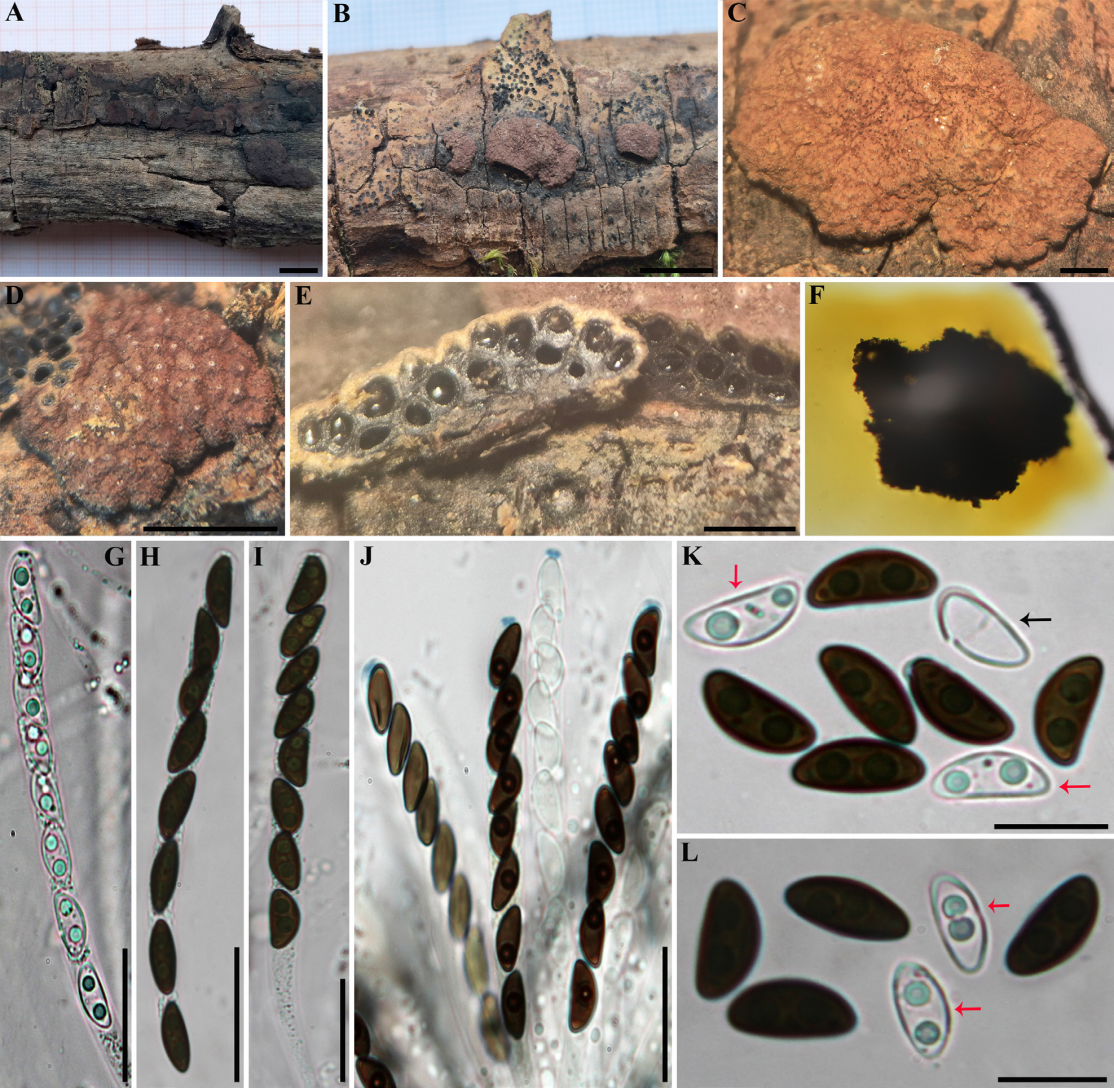
*Hypoxylon perforatum* (GUM 1610). **A, B**. Stromatal habit; **C, D**. Close-up view of stromatal surface; **E**. Stroma in vertical section showing perithecia; **F**. KOH-extractable pigments from stroma; **G**. Immature ascus in water; **H, I**. Mature ascus in water; **J**. Immature and mature asci in Melzer’s reagent; **K, L**. Ascospores in 10% KOH with dehiscent perispore (red arrows: immature ascospores; black arrow: dehiscent perispore). Scale bars: 1 cm (**A, B**); 1 mm (**C–E**); 20 µm (**G–J**); 10 µm (**K, L**).

##### Specimen examined.

Iran • Mazandaran Province, Sisangan County, 36°34'36.89"N, 51°48'37.17"E, 71 m elev., on fallen branch of *Zelkova carpinifolia*, 29 October 2016, M.J. Pourmoghaddam (GUM 1610; living culture MUCL 57728); • Guilan Province, Chaboksar County, Sarvelat forest, 36°57'23"N, 50°32'56"E, 590 m elev., on fallen branch of *Citrus sinensis*, 23 September 2017, M.J. Pourmoghaddam.

##### Notes.

*Hypoxylon perforatum* is one of the *Hypoxylon* species with a cosmopolitan distribution. The KOH-extractable pigments and ostiolar disc with a conspicuous white layer are important diagnostic characters for the species (Sir et al. 2019). A virgariella-like anamorph was originally reported for *H. perforatum* (Ju and Rogers 1996), whereas Raei et al. (2012) and Sir et al. (2019) additionally introduced a nodulisporium-like anamorph. Cedeño-Sanchez et al. (2025) collected a fresh specimen from North Carolina, sequenced, and epitypified the species but could not find its anamorph. The features of the Iranian specimens are in accordance with previous descriptions, except for the inconspicuously to conspicuously striated perispore in 10% KOH, which is seen in specimens from the USA (Ju and Rogers 1996; Sir et al. 2019; Cedeño-Sanchez et al. 2025).

#### Hypoxylon pseudoinvestiens

Taxon classificationFungiXylarialesHypoxylaceae

Pourmoghaddam & C. Lamb. sp. nov.

18892719-B659-55B3-BA29-8F51AD7B49B8

862212

Fig. 16

##### Holotype.

Iran • Guilan Province, Astaneh-ye Ashrafiyeh County, SafraBasteh forest, 37°20'19"N, 49°58'26"E, 14 m elev., on fallen branch of *Quercus castaneifolia*, 3 Oct 2016, M.J. Pourmoghaddam (GUM 1604; living culture MUCL 57729).

##### Etymology.

*Pseudoinvestiens*, referring to the morphological similarity to *H. investiens*.

##### Teleomorph.

Stromata superficial, effused-pulvinate, up to 14 cm long × 0.2–2.5 cm wide, with conspicuous perithecial mounds; surface Dark Vinaceous (82), Dark Brick (60), or Sepia (63) when young, becoming Brown Vinaceous (84), or Chestnut (40); black granules immediately beneath surface and between perithecia, with KOH-extractable pigments Greenish Yellow (16), or Dark Green (21). Perithecia ovoid to cylindrical, 0.25–0.5 mm high × 0.15–0.45 mm wide. Ostioles lower than the stromatal surface. Asci 8-spored, cylindrical, with amyloid, discoid apical apparatus, 0.5–1 µm high × 1.5–2 µm wide, stipe up to 50 µm long, and spore-bearing portion 70–85 × 5–6 µm. Ascospores light brown to brown, unicellular, nearly equilateral, with broadly rounded ends to infrequently narrowly rounded ends, 7–10.5 × 3.5–4 µm, with faint straight germ slit spore-length to slightly less than spore-length on convex side; perispore indehiscent in 10% KOH.

##### Cultures and anamorph.

Colonies on OA covering a 9 cm Petri dish in 2 weeks, at first whitish, becoming Hazel (88) to Isabelline (65), velvety, azonate, with diffuse margins, finally becoming Hazel (88). Conidiogenous structure branching periconiella-like as defined by Ju and Rogers (1996), (Fig. 16L–N). Conidiophores pale brown, finely roughened. Conidiogenous cell hyaline, finely roughened, 11–15 × 2.5–4 µm. Conidia hyaline, smooth, ellipsoid, 3–5 × 2–3.5 µm.

##### Secondary metabolites

**Figure 15.**
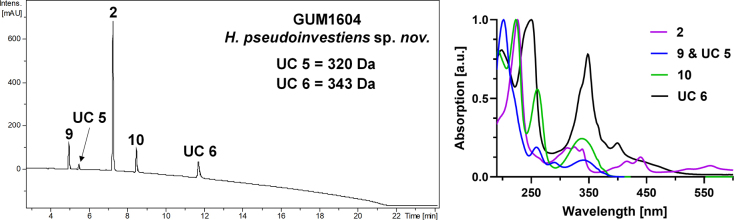
Left: HPLC-UV chromatogram (210 nm) of a stromatal acetone extract of GUM 1604. Right: UV-Vis spectra of designated peaks, revealing daldinone F (9), a potentially dehydroxylated derivative (UC 5), daldinone A (10), and BNT (2), as well as another unknown metabolite (UC 6).

**(see Fig. 15)**. Daldinone F (9) and a potential dehydroxylated derivative (UC 5), daldinone A (10) and BNT (2) and an unknown compound with a mass of 343 Da (UC 6).

**Figure 16.**
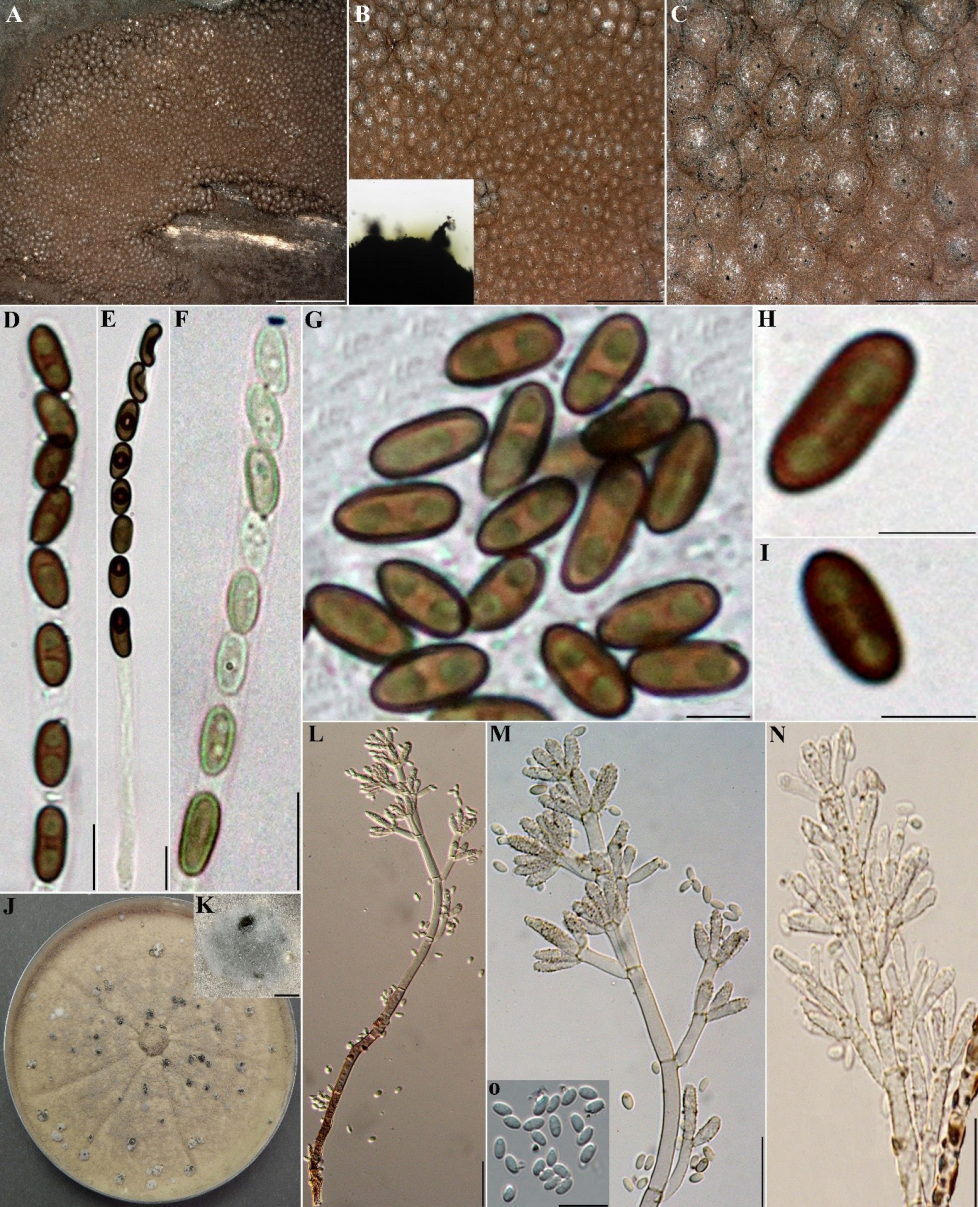
*Hypoxylon pseudoinvestiens* (GUM 1604). **A**. Stromatal habit; **B, C**. Close-up view of stromatal surface, with stromatal pigments in 10% KOH; **D**. Mature ascus in water; **E**. Mature ascus in Melzer’s reagent; **F**. Immature ascus in Melzer’s reagent; **G–I**. Ascospores in water; **J, K**. Surface of colony after 8 weeks of incubation and formation of structures resembling immature stromata; **L–O**. General view of anamorph structure with periconiella-like branching patterns, conidiogenous cells, and conidia. Scale bars: 2.5 mm (**A**); 1.5 mm (**B**); 1 mm (**C**); 20 µm (**D, E**); 10 µm (**F, G**); 5 µm (**H, I**); 1 mm (**K**); 20 µm (**L–N**); 10 µm (**O**).

##### Specimen examined.

Iran • Guilan Province, Rezvanshahr County, 37°37'52"N, 48°02'18"E, 7 m elev., on fallen branch of *Quercus castaneifolia*, 6 October 2016, M.J. Pourmoghaddam (GUM 1605).

##### Notes.

This taxon is phylogenetically close to *H. investienssensu stricto* CBS 118183 (from Malaysia) and YMJ 89062905 (from Taiwan; see Fig. 1). The phylogenetic analyses offer good resolution and statistical support to distinguish these two sister groups in both the MGA and single-locus *tub2* trees (see Suppl. material 1). Most of the characters of the Iranian specimens are largely in accordance with the description by Ju and Rogers (1996). However, the stromata showed conspicuous perithecial mounds, the ascospores are larger [7–10.5 × 3.5–4 vs. (6–)6.5–9.5(–10) × 3–4.5 µm], and variations in the size of conidia [3–5 × 2–3.5 vs. 2.5–3.5 × 2.2–2.8 µm] were observed. In addition, we found typical secondary metabolites daldinone A (**5**), BNT (**2**), and daldinone F (**4**) reported from *H. investiens*, and an additional conspicuous unidentified compound (**UC 6**) with a mass of 343 Da, which has so far never been reported for *H. investiens* (compare also with Hellwig et al. 2005; Bills et al. 2012). The *H. investienssensu stricto*–*H. pseudoinvestiens* clade is the sister group to *H. investienssensu lato*, containing strains MUCL 53307 (from Martinique), STMA 14058 (from Argentina), and CBS 118185 (from Ecuador). There are indications that *H. investiens sensu*Ju and Rogers (1996) represents a species complex that needs to be resolved in the future. Fournier et al. (2015) described further uncultured and unsequenced collections from the French West Indies; they identified them as *H. investiens* based on morphological characters and also reported smaller ascospore ranges when compared to our specimen [(6.8–)7–8(–8.6) × (3.1–)3.3–4.1(–4.5) μm]. Fournier et al. (2015) furthermore noticed additional peculiarities that might necessitate the further segregation of this species complex using polyphasic approaches. Interestingly, our HPLC profiling results on the type material of this taxon [USA: North Carolina, Salem & Pennsylvania, Bethlehem, Syn. 1210 (PH, holotype of *Sphaeria investiens*)] were in full accordance with those reported by Hellwig et al. (2005). Taken together, all evidence justifies the erection of a new species.

*Hypoxylon investienssensu stricto* morphologically resembles *H. dussii* (Fournier et al. 2015), but the most significant feature that distinguishes the former from the latter includes the presence of daldinone A in its stromatal fingerprint (Cedeño-Sanchez et al. 2025). Further similarities of *H. investiens* can be found when compared to *H. pulicicidum*; however, the two can be differentiated by their KOH-extractable pigments (Greenish Yellow or Dark Green vs. dilute Olivaceous Buff), conidiophore structure (periconiella-like vs. nodulisporium-like), stromatal secondary metabolites (presence of daldinone A), and their resolution patterns in molecular phylogenies (see, e.g., Bills et al. 2012).

#### Hypoxylon ticinense

Taxon classificationFungiXylarialesHypoxylaceae

L. E. Petrini apud L. E. Petrini & Müller, Mycol. Helv. 1: 534. 1986

CCC4D5E6-4315-5F58-9FC1-4DF4516731FE

Fig. 18

##### Teleomorph.

Stromata superficial, pulvinate to effused-pulvinate or discoid, with inconspicuous perithecial mounds, up to 4 cm long × 0.4–2.4 cm wide; surface Scarlet (5), Sienna (8) when young, becoming Dark brick (60) to Sepia (63) when mature; orange granules beneath surface and between perithecia, with KOH-extractable pigments Orange (7). Perithecia obovoid to tubular, 0.2–0.35 mm high × 0.15–0.2 mm wide. Ostioles lower than the stromatal surface. Asci 8-spored, cylindrical, with amyloid, discoid apical apparatus, 0.5–0.75 µm high × 1–1.5 µm wide, stipe up to 65 µm long, and spore-bearing portion 40–45 × 3.5–4.5 µm. Ascospores light brown to brown, unicellular, ellipsoid-inequilateral, with narrowly rounded ends, 5–6 × 2–3 µm, with straight germ slit spore-length on convex side; perispore dehiscent in 10% KOH, smooth; epispore smooth.

##### Cultures and anamorph.

Colonies on OA covering a 9 cm Petri dish in 4 weeks, at first whitish, becoming Buff (45), velvety, azonate, with diffuse margins, finally becoming Honey (64). Conidiogenous structure branching virgariella-like as defined by Ju and Rogers (1996). Conidiophores hyaline, smooth. Conidiogenous cell hyaline, smooth, 6.5–12 × 2–3 µm. Conidia hyaline, smooth, ellipsoid, 2.5–3.5 × 1.5–2 µm.

##### Secondary metabolites

**Figure 17.**
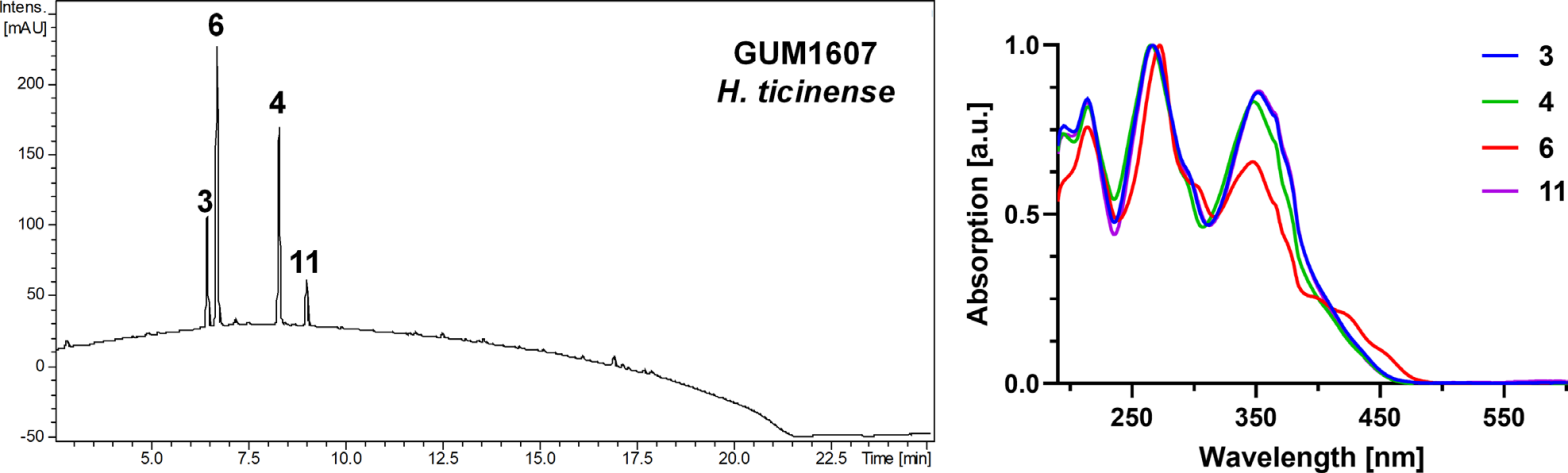
Left: HPLC-UV chromatogram (210 nm) of a stromatal acetone extract of GUM 1607. Right: UV-Vis spectra of designated peaks, revealing mitorubrinol, mitorubrinol acetate, mitorubrinic acid, and mitorubrin (3–4, 6, 11).

**(see Fig. 17)**. Mitorubrinol (3), mitorubrinol acetate (4), mitorubrinic acid (6), and mitorubrin (11).

**Figure 18.**
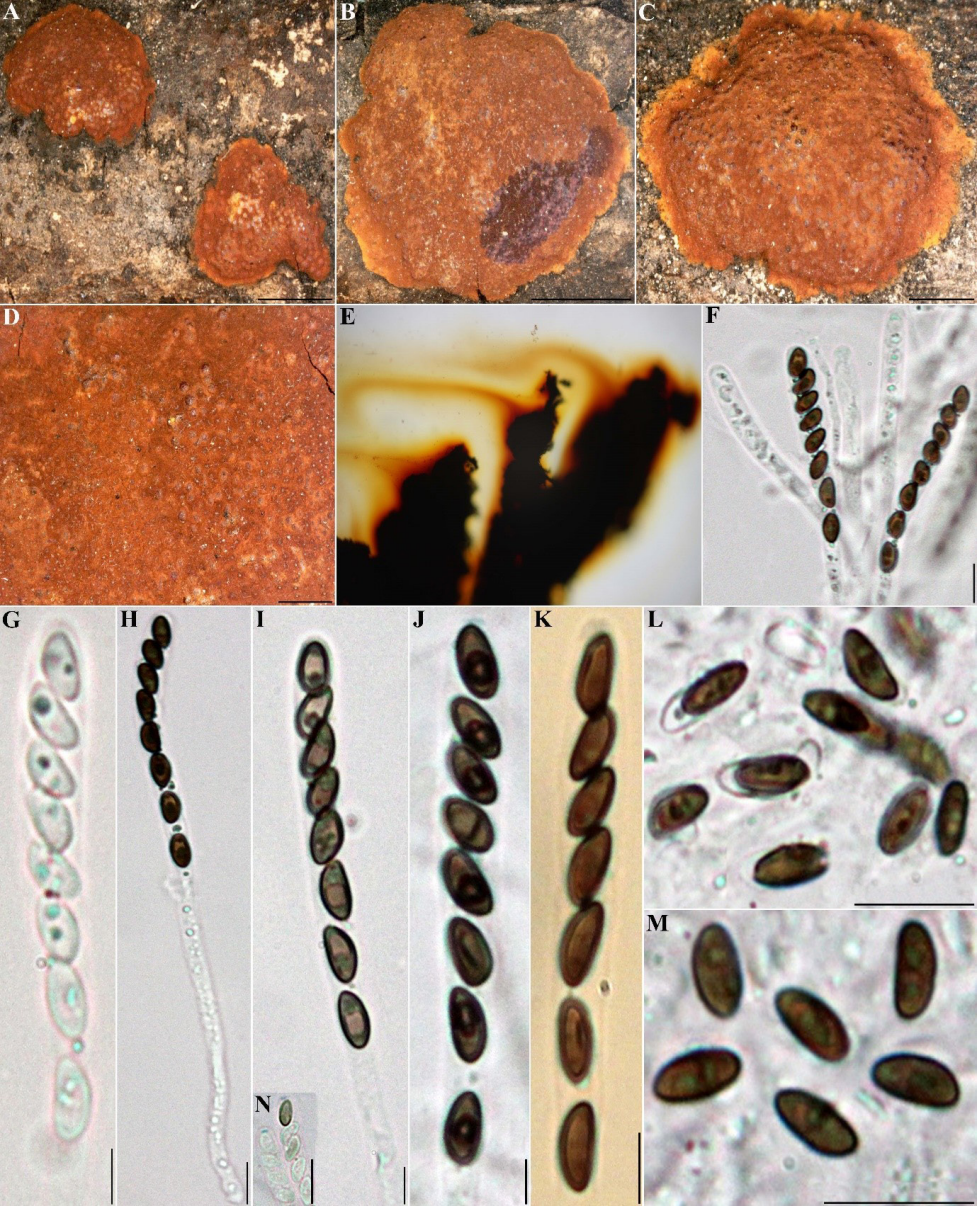
*Hypoxylon ticinense* (GUM 1606). **A–C**. Stromatal habit; **D**. Close-up view of stromatal surface; **E**. Stromatal KOH-extractable pigments; **F**. Immature and mature asci and paraphyses in water; **G**. Immature ascus in water; **H, I**. Mature ascus in water with long stipe; **J, K**. Mature ascus in Melzer’s reagent; **L**. Ascospores in 10% KOH with dehiscent perispore; **M**. Ascospore in water; **N**. Immature ascus tips in Melzer’s reagent. Scale bars: 2.5 mm (**A**); 5 mm (**B**); 1 mm (**C**); 0.5 mm (**D**); 20 µm (**F, H**); 5 µm (**G**); 10 µm (**I–N**).

**Figure 19.**
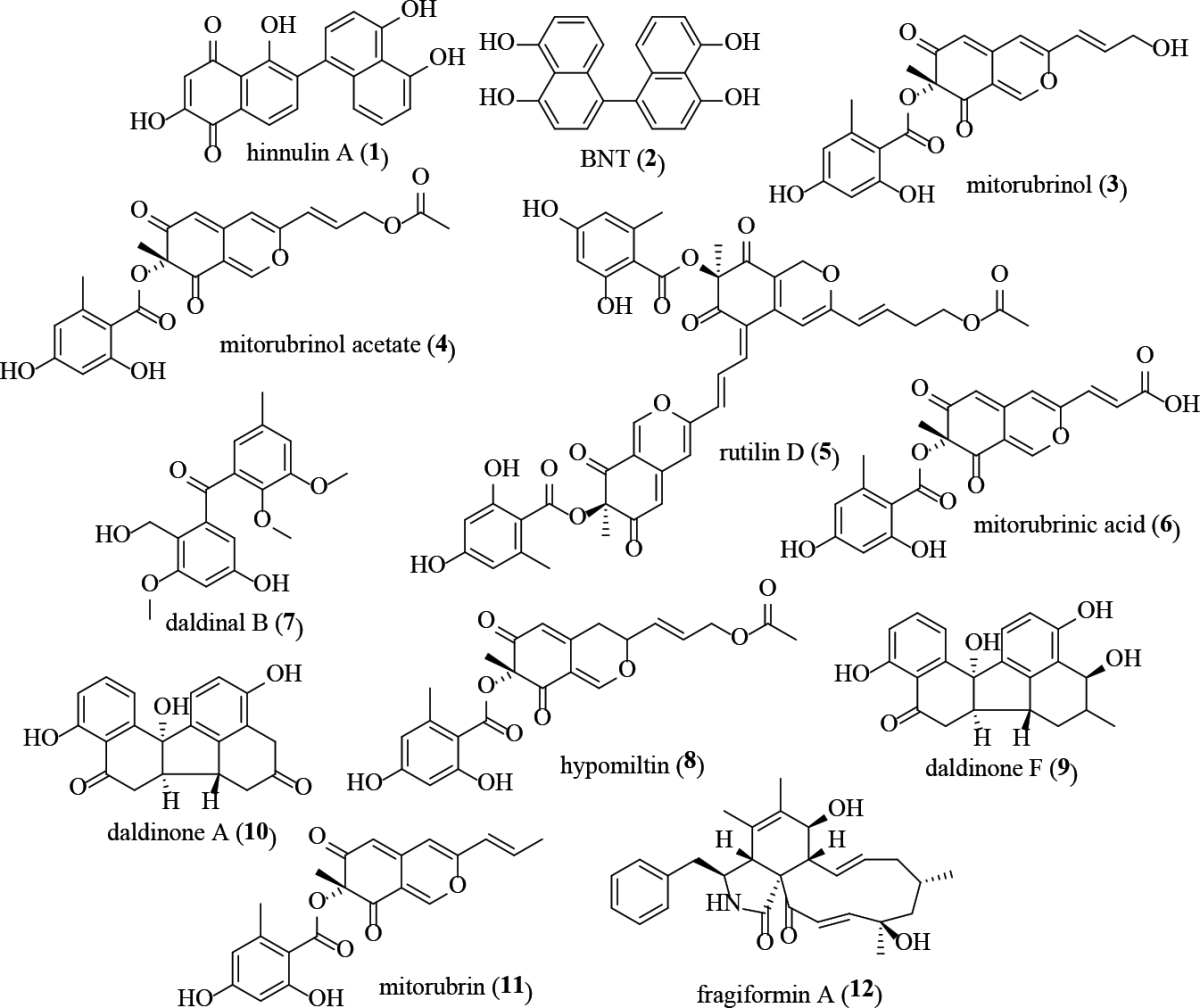
Structures of discussed stromatal secondary metabolites.

##### Specimens examined.

Iran • Guilan Province, Shaft County, Rahimabad (Sefidab) forest, 36°34'11"N, 50°20'26"E, 400 m elev., on fallen branch of *Pterocarya fraxinifolia*, 11 September 2016, leg. M.J. Pourmoghaddam (GUM 1606); Guilan Province, Talesh County, Gisoom forest, 37°47'56"N, 48°49'56"E, 490 m elev., on dead branches (host unknown), 6 October 2016, M.J. Pourmoghaddam (GUM 1607; living culture MUCL 57731).

##### Notes.

This specimen from our collection has similar characters to the type specimen (Ju and Rogers 1996). The stromatal characteristics of this species are strikingly similar to *H. subticinense*. However, the latter differs from *H. ticinense* in having larger ascospores [(7–)8–10(–11) × 4–5 vs. 5–7 × 2.5–3.5 µm], which are ellipsoid, with an indehiscent perispore in KOH, and by some details of anamorph morphology (Ju and Rogers 1996). In addition, both species contain mitorubrin and orsellinic acid as prevailing metabolites, but they are readily distinguished by HPLC analyses. *H. ticinense* lacks the azaphilones rubiginosins A and C and therefore belongs to the fragiforme chemotype but contains mitorubrinol acetate, which is lacking in *H. subticinense* (Stadler et al. 2004; Hellwig et al. 2005).

## Discussion

This study is part of a comprehensive polyphasic investigation of the family Hypoxylaceae in northern Iran, in which we integrated morphological, molecular, and chemotaxonomic datasets to elucidate species boundaries. The occurrence of these taxa in the Hyrcanian forests underscores the region’s role as an ecological refuge and biodiversity hotspot, offering humid temperate microclimates and diverse woody substrates that sustain xylarialean fungi and adding to recent reports of these fungi from northern Iran (Raei et al. 2012, 2014; Hashemi et al. 2014, 2015; Pourmoghaddam et al. 2014, 2018, 2020, 2022a, 2022b, 2023a, 2023b; Lambert et al. 2021, 2025). The combination of multi-locus phylogeny (ITS, LSU, *rpb2*, and *tub2*), detailed morphological characterization, and chemotaxonomy provided robust evidence for the discovery of two new species (*Hypoxylon hyrcanense* and *H. pseudoinvestiens* spp. nov.) and other new records for the Iranian mycobiota. The need for splitting the *H. investiens* complex was already discussed earlier by Fournier et al. (2015), who delimited *H. dussii* from this complex based on substantial variations in morphology and pigmentation. This decision was later corroborated with chemotaxonomic and phylogenetic data by Cedeño-Sanchez et al. (2025). The delimitation of *H. pseudoinvestiens* now renders the clade of *H. investiens* paraphyletic, which heralds further species delimitations in the future. We pursued a similar approach in the *H. fuscum* complex, which eventually led to the delimitation of *H. eurasiaticum* and *H. pseudofuscum* based on polyphasic evidence (Lambert et al. 2021). Similarly, *H. crocopeplum* resolved as polyphyletic in our phylogenetic tree, and the reported phenotypic characters so far render the different collections phenotypically indistinguishable from each other. A key task to resolve these species complexes will involve the selection of appropriate epitypes from North America and, in the best case, involve a study of the contribution of intraspecific variation to their phenotypic diversity.

Our phylogenetic tree is congruent with recent comprehensive analyses of Hypoxylaceae (Cedeño-Sanchez et al. 2023b; Lambert et al. 2025), which demonstrated the paraphyly of *Hypoxylon* and supported the segregation of genera such as *Hypomontagnella* and *Parahypoxylon*. In the phylogenetic tree, *H. hyrcanense* formed a distinct lineage, clearly separated from its sister species *H. ochraceum* and *H. chionostomum*. The final position of members of this clade, however, could not yet be fully resolved. The clustering of *Hypomontagnella submonticulosa* with a European specimen (France) and with Asian specimens reveals a wide ecological amplitude and possibly transcontinental dispersal, which may be linked to host tree distribution in temperate forests. The Iranian *Hypoxylon perforatum* isolate clustered with ex-type isolates of the species and *H. hongheensis*, suggesting that *H. hongheensis* should be treated as a synonym of *H. perforatum*. These phylogenetic relationships highlight biogeographical connections and ultimately allow for taxonomic refinements within the family.

One of the most important contributions of this work lies in demonstrating the advantages of the polyphasic approach for fungal taxonomy. Traditional mycological classification, which relied mainly on morphological and ecological characters, often fails to accurately reflect evolutionary relationships, especially in groups with high morphological convergence such as the *H. rubiginosum* and *H. fuscum* complexes. The integration of molecular phylogenetics with morphology and metabolite profiling enables the discrimination of closely related or cryptic species, provides reproducible and quantifiable data for species identification, and allows the re-evaluation of historical taxa under modern systematic frameworks (Kuhnert et al. 2017; Wendt et al. 2018; Lambert et al. 2021; Cedeño-Sanchez et al. 2025). Moreover, chemotaxonomic data can reveal ecological or functional diversification among lineages, linking taxonomy with secondary metabolism and ecological roles. This multidimensional approach not only improves taxonomic accuracy but also facilitates the discovery of bioactive compounds with pharmaceutical potential (Helaly et al. 2018; Becker and Stadler 2021). Therefore, the present study exemplifies how polyphasic taxonomy bridges classical morphology, modern molecular phylogeny, and analytical chemistry to generate a holistic understanding of fungal biodiversity.

## Supplementary Material

XML Treatment for Annulohypoxylon annulatum

XML Treatment for Hypomontagnella submonticulosa

XML Treatment for Hypoxylon crocopeplum

XML Treatment for Hypoxylon howeanum

XML Treatment for Hypoxylon hyrcanense

XML Treatment for Hypoxylon larissae

XML Treatment for Hypoxylon perforatum

XML Treatment for Hypoxylon pseudoinvestiens

XML Treatment for Hypoxylon ticinense

## Additional information

### Use of AI

No use of AI was reported.

### Author ORCIDs

Mohammad Javad Pourmoghaddam https://orcid.org/0000-0001-6055-7503

Christopher Lambert https://orcid.org/0000-0002-1899-8214

Hermann Voglmayr https://orcid.org/0000-0001-7666-993X

Seyed Akbar Khodaparast https://orcid.org/0000-0002-7480-4170

Marc Stadler https://orcid.org/0000-0002-7284-8671

### Data availability

All of the data that support the findings of this study are available in the main text or Supplementary Information.

## Supplementary materials

Supplementary material 1 Alignment Data type fas https://binary.pensoft.net/file/1586736This dataset is made available under the Open Database License (http://opendatacommons.org/licenses/odbl/1.0/). The Open Database License (ODbL) is a license agreement intended to allow users to freely share, modify, and use this Dataset while maintaining this same freedom for others, provided that the original source and author(s) are credited. Mohammad Javad Pourmoghaddam, Christopher Lambert, Hermann Voglmayr, Seyed Akbar Khodaparast, Marc Stadler
